# Evaluation of a package of continuum of care interventions for improved maternal, newborn, and child health outcomes and service coverage in Ghana: A cluster-randomized trial

**DOI:** 10.1371/journal.pmed.1003663

**Published:** 2021-06-25

**Authors:** Akira Shibanuma, Evelyn Korkor Ansah, Kimiyo Kikuchi, Francis Yeji, Sumiyo Okawa, Charlotte Tawiah, Keiko Nanishi, Sheila Addei, John Williams, Kwaku Poku Asante, Abraham Oduro, Seth Owusu-Agyei, Margaret Gyapong, Gloria Quansah Asare, Junko Yasuoka, Abraham Hodgson, Masamine Jimba, the Ghana EMBRACE Implementation Research Project Team

**Affiliations:** 1 Department of Community and Global Health, Graduate School of Medicine, The University of Tokyo, Tokyo, Japan; 2 Research & Development Division, Ghana Health Service Headquarters, Accra, Ghana; 3 Institute of Health Research, University of Health & Allied Sciences, Ho, Ghana; 4 Department of Health Sciences, Faculty of Medical Sciences, Kyushu University, Fukuoka, Japan; 5 Navrongo Health Research Centre, Navrongo, Upper-East, Ghana; 6 Institute for Global Health Policy Research, Bureau of International Health Cooperation, National Center for Global Health and Medicine, Tokyo, Japan; 7 Kintampo Health Research Centre, Kintampo, Brong-Ahafo, Ghana; 8 Dodowa Health Research Centre, Dodowa, Greater Accra, Ghana; 9 Research and Education Center for Prevention of Global Infectious Diseases of Animals, Tokyo University of Agriculture and Technology, Tokyo, Japan; London School of Hygiene and Tropical Medicine, UNITED KINGDOM

## Abstract

**Background:**

In low- and middle-income countries (LMICs), the continuum of care (CoC) for maternal, newborn, and child health (MNCH) is not always complete. This study aimed to evaluate the effectiveness of an integrated package of CoC interventions on the CoC completion, morbidity, and mortality outcomes of woman–child pairs in Ghana.

**Methods and findings:**

This cluster-randomized controlled trial (ISRCTN: 90618993) was conducted at 3 Health and Demographic Surveillance System (HDSS) sites in Ghana. The primary outcome was CoC completion by a woman–child pair, defined as receiving antenatal care (ANC) 4 times or more, delivery assistance from a skilled birth attendant (SBA), and postnatal care (PNC) 3 times or more. Other outcomes were the morbidity and mortality of women and children. Women received a package of interventions and routine services at health facilities (October 2014 to December 2015). The package comprised providing a CoC card for women, CoC orientation for health workers, and offering women with 24-hour stay at a health facility or a home visit within 48 hours after delivery. In the control arm, women received routine services only. Eligibility criteria were as follows: women who gave birth or had a stillbirth from September 1, 2012 to September 30, 2014 (before the trial period), from October 1, 2014 to December 31, 2015 (during the trial period), or from January 1, 2016 to December 31, 2016 (after the trial period). Health service and morbidity outcomes were assessed before and during the trial periods through face-to-face interviews. Mortality was assessed using demographic surveillance data for the 3 periods above. Mixed-effects logistic regression models were used to evaluate the effectiveness as difference in differences (DiD). For health service and morbidity outcomes, 2,970 woman–child pairs were assessed: 1,480 from the baseline survey and 1,490 from the follow-up survey. Additionally, 33,819 cases were assessed for perinatal mortality, 33,322 for neonatal mortality, and 39,205 for maternal mortality. The intervention arm had higher proportions of completed CoC (410/870 [47.1%]) than the control arm (246/620 [39.7%]; adjusted odds ratio [AOR] for DiD = 1.77; 95% confidence interval [CI]: 1.08 to 2.92; *p* = 0.024). Maternal complications that required hospitalization during pregnancy were lower in the intervention (95/870 [10.9%]) than in the control arm (83/620 [13.4%]) (AOR for DiD = 0.49; 95% CI: 0.29 to 0.83; *p* = 0.008). Maternal mortality was 8/6,163 live births (intervention arm) and 4/4,068 live births during the trial period (AOR for DiD = 1.60; 95% CI: 0.40 to 6.34; *p* = 0.507) and 1/4,626 (intervention arm) and 9/3,937 (control arm) after the trial period (AOR for DiD = 0.11; 95% CI: 0.11 to 1.00; *p* = 0.050). Perinatal and neonatal mortality was not significantly reduced. As this study was conducted in a real-world setting, possible limitations included differences in the type and scale of health facilities and the size of subdistricts, contamination for intervention effectiveness due to the geographic proximity of the arms, and insufficient number of cases for the mortality assessment.

**Conclusions:**

This study found that an integrated package of CoC interventions increased CoC completion and decreased maternal complications requiring hospitalization during pregnancy and maternal mortality after the trial period. It did not find evidence of reduced perinatal and neonatal mortality.

**Trial registration:**

The study protocol was registered in the International Standard Randomised Controlled Trial Number Registry (90618993).

## Abstract 2

Akira Shibanuma and co-workers study a package of maternal and child health interventions in a cluster-randomized trial done in Ghana.

## Author summary

**Why was this study done?:**

- High individual coverages of antenatal care (ANC), delivery assistance from a skilled birth attendant (SBA), and postnatal care (PNC) do not mean that woman–child pairs receive all these services along the continuum of care (CoC) in maternal, newborn, and child health (MNCH).
- To our knowledge, no previous study has evaluated the effectiveness of interventions on CoC completion (or receiving all the key services along the CoC) under a real-world setting.

**What did the researchers do and find?:**

- We conducted a cluster-randomized controlled trial to evaluate the effectiveness of an integrated package of CoC interventions in a variety of healthcare settings and health facilities at 3 Health and Demographic Surveillance System (HDSS) sites in Ghana.
- An integrated package of interventions that aimed at enhancing CoC in MNCH increased CoC completion and decreased maternal complications requiring hospitalization during pregnancy. It also reduced maternal mortality after the trial period, although evidence of decreasing perinatal and neonatal mortality was not found.
- We found strong spillover of intervention effectiveness among mothers and children living in subdistricts in the control arm.

**What do these findings mean?:**

- CoC completion among mothers and children can be improved through a package of interventions combining the use of inexpensive home-based records, orientation for health workers, and encouragement for retention to postpartum care under a real-world setting.
- CoC completion can be accelerated through better health systems components, such as more community-based health services and upgrading human resources for the health sector.

## Background

Mortality related to pregnancy and childbirth remains a major public health concern in low- and middle-income countries (LMICs) [1,2]. Globally, in 2017, 99% of maternal deaths occurred in these countries [1]. Furthermore, in 2018, LMICs accounted for 98% of all infant deaths worldwide [2]. Despite the efforts made toward achieving the Millennium Development Goals for reducing maternal and child mortality [3], many countries have yet to meet this target. Thus, this continues to be one of the 13 targets of the Sustainable Development Goals (SDGs) 3.

In the field of maternal, newborn, and child health (MNCH), the continuum of care (CoC) is a widely shared policy goal among LMICs governments and their development partners. The CoC addresses the importance of care from prepregnancy to motherhood and childhood (time dimension) and from the community to higher-tier health facilities (place dimension) [4–6]. It aims to ensure that every woman and child receives timely, quality care [4,7]. Under the time dimension, women are expected to receive antenatal care (ANC) and give birth with assistance from a skilled birth attendant (SBA) [5]; additionally, women and children are expected to receive postnatal care (PNC) at scheduled times [8–10].

However, high individual coverage of these interventions, namely ANC, SBA delivery, and PNC, does not mean that woman–child pairs receive them all [11,12]. To improve the CoC, women should be informed during early pregnancy about the importance of receiving subsequent care along the CoC. Particularly, women with pregnancy complications should be screened and instructed to receive necessary care during pregnancy [8,13–16]. For this screening and instruction, some LMICs use different tools, such as home-based records (e.g., maternal and child health handbooks), to record one’s health services access history [17–20]. Health promotion starting from the prepregnancy stage could improve perinatal and neonatal mortality [6]. However, no study to date has evaluated the effectiveness of interventions developed to ensure access to ANC, SBA delivery, or PNC under a real-world setting.

Ghana has demonstrated an extensive improvement in MNCH health indicators, although the current trend of improvement might not ensure that the global targets set forth in the SDGs are achieved, particularly for maternal health [21–23]. In Ghana, at the time this study began, maternal mortality ratio was 319 out of 100,000 live births in 2015, although the country’s target in 2015 was 190. Likewise, under-five mortality rate was 59 out of 1,000 live births in 2016, although the country’s target in 2015 was 42 [21]. CoC completion rate among woman–child pairs, or the coverage of receiving all of ANC 4 times, SBA delivery, and PNC 3 times, was 8%, while the coverage of ANC alone was 86% and that of SBA delivery alone was 76% in 2014 [11].

In Ghana, the coverage of PNC within 24 hours after delivery was substantially low (25%), compared with the coverage of ANC 4 times or more and SBA delivery [11]. PNC can be provided either at a health facility or by community outreach in women’s homes [24–26]. Women and newborns can receive PNC at a health facility if they are retained there longer after delivery [24]. The retention is part of Ghana’s MNCH guidelines. The coverage of PNC can also be improved by providing PNC at women’s homes, although an adequate number of trained community health workers would be necessary to conduct timely home visits [25–27]. In Ghana, community health workers are designated to conduct home visits [28]. Nonetheless, prior to this study, retention and home visits had yet to be largely adhered to in the study area, mainly owing to the lack of physical infrastructure and human resources.

The Ghana EMBRACE Implementation Research Project implemented a package of interventions aimed at enhancing the CoC among women during pregnancy, delivery, and postpartum stages in Ghana. Therefore, this study aimed to evaluate the effectiveness of a package of CoC interventions on health service, morbidity, and mortality outcomes related to MNCH at 3 Health and Demographic Surveillance System (HDSS) sites in Ghana.

## Methods

### Study design

Using an effectiveness–implementation hybrid design, this study was implemented as a cluster-randomized controlled trial. As explained in the protocol paper [29], this trial was conducted to evaluate the effectiveness of an integrated package of CoC interventions and the process of implementing the package in a variety of healthcare settings and health facilities that provided MNCH services in Ghana (see S1 Protocol and S1 CONSORT Checklist). This study was conducted to show the results of the effectiveness of the interventions. The trial was conducted at 3 HDSS sites in Ghana: Dodowa, Kintampo, and Navrongo. These sites had 6 districts and 36 subdistricts in total. In 2011, based on administrative data, the total population of the 3 sites was 469,000, and the total number of deliveries was estimated to be 14,539 annually. The latter number was calculated based on an assumption of a crude birth ratio of 3.1 per 100 persons.

In the study area, different types of health facilities were in operation, including public hospitals, health centers, community-based health facilities—called community-based health planning and services (CHPS) in Ghana—and private health facilities. CHPS was designed to provide primary care services at the community level.

### Participants

To evaluate mortality outcomes, women were selected according to the following inclusion criteria: those at reproductive age (i.e., ranging from 15 to 49 years), who lived in the study area, and who gave birth or had a stillbirth before the trial period (from September 1, 2012 to September 30, 2014), during the trial period (from October 1, 2014 to December 31, 2015), and after the trial period (from January 1, 2016 to December 31, 2016).

To evaluate health service and morbidity outcomes, women were selected according to the following inclusion criteria: those who lived in the study area and gave birth or had a stillbirth before the survey period for the baseline survey (conducted from July 1, 2014 to September 30, 2014). For the follow-up survey, the inclusion criteria were as follows: those who lived in the study area and received ANC, gave birth, or received PNC during the trial period (conducted from October 1, 2015 to December 31, 2015).

Written informed consent was obtained from all women who participated in the surveys. Their participation was voluntary, and their confidentiality was secured. However, this study did not collect informed consent from women who received parts of the interventions as routine MNCH services in real-world conditions, although they received information about the study.

### Randomization and masking

The unit for intervention allocation was the subdistrict, which refers to the smallest health administration unit in Ghana. Among the 36 subdistricts at the 3 collaborating HDSS sites, 32 were included. The remaining 4 were excluded as there were plans for another MNCH project to be conducted. Since the number of subdistricts might have been too small to ensure a balance of subdistrict characteristics between the intervention and control arms, subdistricts were matched by dividing the 32 subdistricts into 16 pairs. The pairs were matched based on population, the number of deliveries in each cluster, and the number of midwives available. A subdistrict was immediately allocated as an intervention arm if it had the only district hospital in a district.

This method to allocate the intervention arm was used because women had autonomy in health facility choice in the study area, and many women were expected to choose the district hospital, regardless of their arms, especially for delivery. The district hospital also served as the referral hospital for the whole district. In addition, it played a leadership role in managing MNCH services at the district level. In the other pairs, each pair had 1 subdistrict that was randomly allocated as part of the intervention arm; this procedure was performed by a data analyst who was not a primary investigator. Based on the nature of the interventions in this study, masking did not take place among women and health workers.

### Interventions

The integrated package of CoC interventions was designed based on the analyses of formative studies [11–13,30–32] and consultations with health administrators (i.e., at the national and subnational levels), health workers, and mothers.

In the intervention arm, the following activities took place in addition to routine MNCH services: the distribution and use of the CoC card (A-1); CoC orientation for health workers (A-2); 24-hour retention of women and newborns at a health facility after delivery (B-1); and PNC by home visits (B-2). Women in the control arm received routine MNCH services only. For A-1, when women received care in a health facility, they received the 1-page CoC card, which was designed to schedule subsequent visits to receive ANC, SBA delivery, and PNC [33]. CoC card was also used to record the visiting history, the receipt of key care components during ANC and PNC, and complications. Moreover, it has pictorial images for services and key care components, drawn by Ghanaian artists under the supervision and testing by Ghanaian researchers. This was done to ensure that women and other household members who saw the CoC card could easily understand the images.

For A-2, health workers at health facilities in the intervention arm received training on CoC services and the package of CoC interventions used in this study. For B-1, women were encouraged to stay with their children at the health facility for at least 24 hours after delivery. For B-2, community health officers were encouraged to visit women’s homes within 48 hours after delivery when they delivered in their homes. Based on the formative research of the Ghana EMBRACE Implementation Research Project [11,12], B-1 and B-2 were designed to address the relatively low coverage of PNC within 2 days after delivery.

### Sampling of women and children

To evaluate health service and morbidity outcomes, 1,500 women were randomly selected from the 3 sites during the baseline survey period, which occurred before the trial period (from July to September 2014). For the follow-up survey, randomized selection was repeated (i.e., 1,500 women from the 3 sites), but this occurred at the end of the trial period (from October to December 2015). The sampling frame was obtained from a list of pregnant women that the 3 sites maintained. The sample size calculation was based on results from the formative research [11], in which the CoC completion rate was 8%. This study was designed with a targeted CoC completion rate of 60%. Under this scenario, based on the results of the formative study, it was expected that the coverage of ANC 4 times or more would increase from 86.6% to 95.0% with an intraclass correlation coefficient of 0.02675 (average cluster size was 102). A detailed explanation of this process is provided in the protocol paper [29].

In this study, to evaluate mortality outcomes, pregnancy and birth outcome registration data at the HDSS sites in Kintampo and Navrongo were used; however, registration data from Dodowa were not used, as mortality data were incomplete. In Kintampo and Navrongo, key informants identified pregnancies at the community level. Based on the assumption of a 25% reduction in perinatal mortality rate (from 31 to 23 per 1,000 pregnancies) and an intraclass correlation coefficient of 0.0007256 (average cluster size was 102), the required sample size was determined to be 15,000 each before, during, and after the trial.

In the baseline survey, out of a total of 602 communities (administrative units within subdistricts), 146 were randomly selected as the primary sampling units (PSUs). The number of PSUs in a subdistrict was determined according to the probability proportionate to the population. The number of eligible women differed by PSU, with 41.7 eligible women per selected PSU, on average. Then, from each PSU, and based on the pregnancy registries at each of the 3 sites, 10 eligible women were randomly selected. The same communities were selected for the follow-up survey; however, when the number of eligible women in a selected community was less than 10, a neighboring community was surveyed.

### Data collection

In the baseline and follow-up surveys for health service and morbidity outcomes, trained interviewers conducted face-to-face interviews with randomly selected women at the 3 sites. The questionnaire was developed mainly based on items from the Demographic and Health Survey in Ghana [34], such as demographic and socioeconomic characteristics, MNCH services uptake, health complications (during the pregnancy, delivery, and postnatal periods), pregnancy outcomes, and care-seeking behaviors.

### Outcomes

In the study, the primary outcome was CoC completion. A woman–child pair was considered to have met the criteria for CoC completion when they received all of the following: ANC 4 times or more; delivery assistance from a SBA at a health facility; and PNC within 48 hours, around 1 week, and around 6 weeks after delivery. This study analyzed CoC completion rate as the percentage of woman–child pairs who met the criteria for CoC completion by arm before and during the trial periods. The following other outcomes were also measured: the coverage of PNC within 48 hours of delivery, the prevalence of complications that required hospitalization for 24 hours or more among women and children, and all causes of maternal, perinatal, and neonatal mortality.

### Statistical analysis

In this study, intervention effectiveness was estimated using the intention-to-treat approach. This study used Rao–Scott cluster-adjusted chi-squared tests to examine outcome differences. In addition, difference in differences (DiD) rate ratios and absolute risk differences were reported. It also used mixed-effects logistic regression models with a random intercept at the HDSS site and community levels to estimate the effectiveness of the intervention on the outcomes. Mixed-effects models were used, instead of generalized estimating equations as stated in the protocol paper, as the model accounting for random effects at the 2 levels (HDSS site and community). To present the absolute size of the effectiveness, number needed to treat (NNT) was also estimated for statistically significant results based on mixed-effect linear regression models. Regarding the health service and morbidity outcomes, pooled data at baseline and the follow-up surveys were used. That is, intervention effectiveness was estimated as DiD. The regression models included the confounders listed below to adjust for possible differences in the characteristics between the intervention and control arms. Regarding mortality outcomes, pooled data were used before, during, and after the trial periods without controlling for confounders because of unavailability in HDSS data. Intervention effectiveness was estimated using the 2 interaction terms (arm and during the trial period; arm and after the trial period). An analysis of missing data was not performed for health service and morbidity outcomes because the woman–child pairs surveyed did not have missing information for the outcomes or covariates. Additionally, this analysis was not performed for mortality assessment as the assessment did not have information on missing observations, and covariates were not available.

The main variable of interest in this study was living in a subdistrict in the intervention arm. In the regression models, the interaction between living in a subdistrict in the intervention arm (versus control arm) and follow-up period (versus baseline period) captured the effectiveness of the intervention as a DiD indicator. Supplementary analyses were performed to investigate the differences in improvements of CoC completion by the characteristics of the nearest health facilities. Moreover, to examine the differences in improvements of the CoC completion by the level of development of the health systems at the subdistrict level, this study conducted subsample analyses; to do so, it stratified the dataset using the following characteristics: whether people lived in a subdistrict far from the main road or not, in a subdistrict that had public hospitals and health centers, in a subdistrict with a higher density of health facilities (per 100 pregnancy cases a year) or not, and in a subdistrict where there is a midwife stationed in at least 1 health facility.

### Ethical considerations

In Ghana, this study was approved by the Ethics Review Committee of Ghana Health Service (GHS; reference: GHS-ERC: 13/03/14) and the Institutional Review Boards of the Dodowa Health Research Centre (reference: FGS-DHRC: 280214), the Kintampo Health Research Centre (reference: 2014–11), and the Navrongo Health Research Centre (reference: NHRCIRB137). In Japan, it was approved by the Research Ethics Committee of the University of Tokyo (reference serial number: 10513). The study protocol was registered with the International Standard Randomized Controlled Trial Number Registry (90618993).

## Results

**Fig 1:**
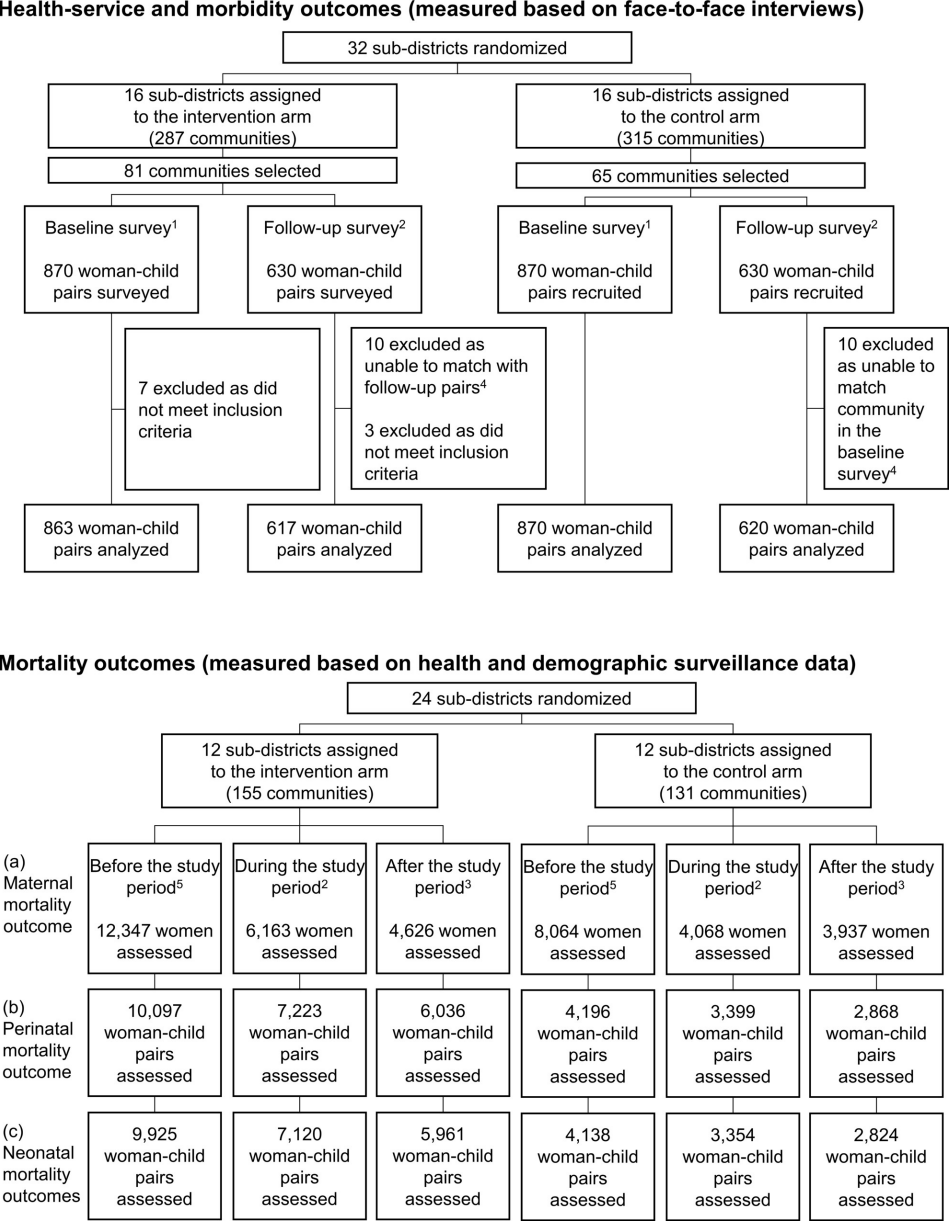
Woman–child pairs included in this study. ^1^Women gave birth or had a stillbirth before the trial period (from September 1, 2012 to June 30, 2014). ^2^Women gave birth or had a stillbirth during the trial period (from October 1, 2014 to December 31, 2015). ^3^Women gave birth or had a stillbirth after the trial period (from January 1, 2016 to December 31, 2016). ^4^A community where 10 woman–child paired were recruited at the baseline survey had been replaced with a neighboring community as it had less than 10 eligible women at the follow-up survey. However, after the survey, we excluded these communities from the analysis as we concluded that they had substantially different socioeconomic characteristics. ^5^Women gave birth or had a stillbirth before the trial period (from September 1, 2012 to September 30, 2014).

Fig 1 presents the number of woman–child pairs included in this study. In total, 2,970 woman–child pairs were assessed (1,480 from the baseline survey and 1,490 from the follow-up survey) for service-related and morbidity outcomes. Among the 1,500 pairs each initially recruited in the baseline and follow-up surveys, 30 pairs were excluded due to the following reasons. Among them, 10 pairs were excluded in the baseline survey because they did not meet the inclusion criteria. Additionally, 10 pairs in one community in the baseline survey and 10 pairs in its neighboring community in the follow-up survey were excluded. This was because the community excluded at baseline did not have the sufficient number of women in the follow-up survey, and its neighboring community was later judged to have substantially different characteristics from the original community in the baseline survey. This study assessed perinatal mortality outcome from 33,819 cases, neonatal mortality outcome from 33,322 cases, and maternal mortality outcome from 39,205 cases recorded in the HDSS database.

Table 1 presents the number of health facilities in the study area. Health facilities were excluded if no women from the baseline or follow-up surveys visited for ANC, delivery, or the first PNC. Women received ANC, SBA delivery, and PNC in 109 health facilities. In the intervention arm, among the 56 health facilities where women received ANC, 11 provided all 4 interventions described in the Methods section. From these health facilities, 84.6% of women and children in the follow-up survey received the package of CoC interventions.

**Table 1:** Number of health facilities by type and content of interventions.

	Public hospital	Health center	CHPS	Private	Total
Intervention arm					
Distribution of CoC card (A-1) and CoC orientation (A-2) only	1	0	0	4	5
A-1, A-2, and 24-hour retention as inpatients at a health facility (B-1)	2	0	0	1	3
A-1, A-2, and PNC by home visit (B-2)	0	2	35	0	37
All interventions	0	5	6	0	11
Control area	2	9	37	5	53

Note: Health facilities reported above were those which participated in this study. Part of private health facilities in the study area did not participate in this study.

CHPS, community-based health planning and services; CoC, continuum of care; PNC, postnatal care.

Table 2 summarizes the health service and morbidity outcomes. CoC completion rate at follow-up showed a greater increase (compared with the rates at baseline) in the intervention arm (from 7.5% to 47.1%) than in the control arm (from 9.2% to 39.7%) (*p* = 0.031). In the DiD analysis, CoC completion rate improved by 46% or 9.2 percentage points. Among the components of CoC completion, PNC 3 times at follow-up was greater in the intervention arm (57.9%) than the control arm (51.1%) (*p* = 0.044), while it was almost identical between the arms at baseline (11.2% versus 11.5%). In the DiD analysis, it increased by 16% or 7.1 percentage points.

**Table 2:** Health service and morbidity outcomes of this study.

	Before the trial period					During the trial period						
	Intervention (*n* = 863)		Control (*n* = 617)			Intervention (*n* = 870)		Control (*n* = 620)			RR^†^	ARD^‡^
	*n*	(%)	*n*	(%)	*p*-value^¶^	*n*	(%)	*n*	(%)	*p*-value^¶^		
Completed CoC	65	(7.5)	57	(9.2)	0.384	410	(47.1)	246	(39.7)	0.031	1.46	9.2
ANC 4 times or more	590	(68.4)	415	(67.3)	0.703	669	(76.9)	479	(77.3)	0.900	0.98	−1.5
Delivered with assistance by a SBA	631	(73.1)	463	(75.0)	0.620	713	(82.0)	496	(80.0)	0.559	1.05	3.9
Received PNC within 48 hours, around 1 week, and around 6 weeks	97	(11.2)	71	(11.5)	0.911	504	(57.9)	317	(51.1)	0.044	1.16	7.1
PNC within 48 hours	465	(53.9)	321	(52.0)	0.670	706	(81.1)	498	(80.3)	0.777	0.98	−1.0
PNC within 48 hours by home visits	4	(0.5)	1	(0.2)	0.350	74	(8.5)	56	(9.0)	0.779	0.33	−0.8
Maternal complications during pregnancy	309	(35.8)	217	(35.2)	0.830	322	(37.0)	235	(37.9)	0.780	0.96	−1.5
of which required hospitalization	102	(11.8)	46	(7.5)	0.014	95	(10.9)	83	(13.4)	0.269	0.51	−6.8
Maternal complications during and immediately after the delivery	120	(13.9)	97	(15.7)	0.493	111	(12.8)	85	(13.7)	0.592	1.05	0.9
of which required hospitalization	91	(10.5)	87	(14.1)	0.150	81	(9.3)	61	(9.8)	0.719	1.27	3.0
Maternal complications within 6 weeks of delivery	96	(11.1)	82	(13.3)	0.315	68	(7.8)	50	(8.1)	0.862	1.16	1.9
of which required hospitalization	15	(1.7)	10	(1.6)	0.848	18	(2.1)	14	(2.3)	0.820	0.85	−0.3
Child’s danger signs within 6 weeks of delivery	131	(15.2)	125	(20.3)	0.035	104	(12.0)	97	(15.6)	0.079	1.02	1.4
of which required hospitalization	29	(3.4)	18	(2.9)	0.645	37	(4.3)	23	(3.7)	0.617	1.00	0.1

^†^RR during the trial period divided by RR before the trial period.

^‡^Risk difference before the trial period subtracted from risk difference during the trial period.

^¶^Rao–Scott cluster-adjusted chi-squared test was used to compare the proportions of an outcome by arm.

ANC, antenatal care; ARD, absolute risk difference; CoC, continuum of care; PNC, postnatal care; RR, rate ratio; SBA, skilled birth attendant.

As shown in Table 3, no significant baseline differences in mortality outcomes were detected between the arms. Maternal mortality was 8 cases among 6,163 live births in the intervention arm and 4 among 4,068 live births in the control arm during the trial period (*p* = 0.655; in the DiD analysis, maternal mortality ratio increased by 60% or equivalent to 64 cases per 100,000 live births) and 1 among 4,626 in the intervention arm and 9 among 3,937 in the control arm after the trial period (*p* = 0.007; in the DiD analysis, maternal mortality ratio decreased by 89% or equivalent to 175 cases per 100,000 live births). Perinatal mortality was 130 cases among 6,036 live and stillbirths in the intervention arm and 104 among 4,196 in the control arm during the trial period (*p* = 0.303; in the DiD analysis, perinatal mortality rate decreased by 19% or equivalent to 5 case per 1,000 births) and 71 among 3,399 in the intervention arm and 64 among 2,868 in the control arm after the trial period (*p* = 0.745; in the DiD analysis, perinatal mortality rate decreased by 13% or equivalent to 3 cases per 1,000 births). Neonatal mortality was 73 cases among 5,961 live births in the intervention arm and 57 among 4,138 in the control arm during the trial period (*p* = 0.510; in the DiD analysis, neonatal mortality rate decreased by 3% or equivalent to 0 case per 1,000 live births) and 30 among 3,354 in the intervention arm and 25 among 2,824 in the control arm after the trial period (*p* = 0.970; in the DiD analysis, neonatal mortality rate increased by 11% or 2 cases per 1,000 live births).

**Table 3:** Mortality outcomes of this study.

	Before the trial period					During the trial period							After the trial period						
	Intervention (*n* = 10,097)		Control (*n* = 7,223)			Intervention (*n* = 6,036)		Control (*n* = 4,196)			RR^§^	ARD^#^	Intervention (*n* = 3,399)		Control (*n* = 2,868)			RR^§^	ARD^#^
	*n*	Rate^†^	*n*	Rate^†^	*p*-value^††^	*n*	Rate^†^	*n*	Rate^†^	*p*-value^††^			*n*	Rate^†^	*n*	Rate^†^	*p*-value^††^		
Perinatal mortality	288	(28.5)	192	(26.6)	0.512	130	(21.5)	104	(24.8)	0.303	0.81	−5.2	71	(20.9)	64	(22.3)	0.745	0.87	−3.4
Stillbirth	172	(17.0)	103	(14.3)	0.174	75	(12.4)	58	(13.8)	0.511	0.75	−4.2	45	(13.2)	44	(15.3)	0.615	0.72	−4.9
	Intervention (*n* = 9,925)		Control (*n* = 7,120)			Intervention (*n* = 5,961)		Control (*n* = 4,138)			RR^§^	ARD^#^	Intervention (*n* = 3,354)		Control (*n* = 2,824)			RR^§^	ARD^#^
	*n*	Rate^‡^	*n*	Rate^‡^	*p*-value^††^	*n*	Rate^‡^	*n*	Rate^‡^	*p*-value^††^			*n*	Rate^‡^	*n*	Rate^‡^	*p*-value^††^		
Neonatal mortality	144	(14.5)	113	(15.9)	0.512	73	(12.2)	57	(13.8)	0.510	0.97	−0.2	30	(8.9)	25	(8.9)	0.970	1.11	1.5
Early neonatal mortality	116	(11.7)	89	(12.5)	0.665	55	(9.2)	46	(11.1)	0.397	0.89	−1.1	26	(7.8)	20	(7.1)	0.768	1.17	1.5
Late neonatal mortality	28	(2.8)	24	(3.4)	0.533	18	(3.0)	11	(2.7)	0.734	1.36	0.9	4	(1.2)	5	(1.8)	0.530	0.80	0.0
	Intervention (*n* = 12,347)		Control (*n* = 8,064)			Intervention (*n* = 6,163)		Control (*n* = 4,068)			RR^§^	ARD^#^	Intervention (*n* = 4,626)		Control (*n* = 3,937)			RR^§^	ARD^#^
	*n*	Ratio^¶^	*n*	Ratio^¶^	*p*-value^††^	*n*	Ratio^¶^	*n*	Ratio^¶^	*p*-value^††^			*n*	Ratio^¶^	*n*	Ratio^¶^	*p*-value^††^		
Maternal mortality	19	(153.9)	15	(186.0)	0.608	8	(129.8)	4	(98.3)	0.655	1.60	63.6	1	(21.6)	9	(228.6)	0.007	0.11	−174.9

^†^Per 1,000 live and stillbirths.

^‡^Per 1,000 live births.

^¶^Per 100,000 live births.

^§^RR during (or after) the trial period divided by RR before the trial period.

^#^Risk difference before the trial period subtracted from risk difference during (or after) the trial period.

^††^Rao–Scott cluster-adjusted chi-squared test was used to compare the proportions of an outcome by arm.

ARD, absolute risk difference; RR, rate ratio.

Table 4 shows the background characteristics of women, children, and their households for the health service and morbidity outcomes. Regardless of the arm, and compared with women in the baseline survey, women in the follow-up survey tended to be younger, more educated, less in the formal marriage (more cohabitating, divorced, separated, widowed, or never married), have joined the health insurance scheme, and have a more educated partner. In the follow-up survey, socioeconomic status differed significantly by arm (*p* < 0.001).

**Table 4:** Characteristics of women and their partners for the baseline and follow-up surveys.

	Baseline					Follow-up				
	Intervention (*n* = 863)		Control (*n* = 617)			Intervention (*n* = 870)		Control (*n* = 620)		
	*n*	%	*n*	%	*p*-value^†^	*n*	%	*n*	%	*p*-value^†^
Age (mean, SD)	28.5	(6.8)	28.6	(6.6)	0.906	26.4	(6.5)	26.5	(6.6)	0.797
Education					0.859					0.296
Did not complete primary	257	(29.8)	178	(28.8)		182	(20.9)	145	(23.4)	
Completed primary	222	(25.7)	170	(27.6)		242	(27.8)	196	(31.6)	
Completed secondary	289	(33.5)	209	(33.9)		326	(37.5)	207	(33.4)	
Above secondary	95	(11.0)	60	(9.7)		120	(13.8)	72	(11.6)	
Parity					0.810					0.371
None or once	196	(22.7)	128	(20.7)		299	(34.4)	187	(30.2)	
Twice or thrice	323	(37.4)	243	(39.4)		335	(38.5)	249	(40.2)	
Four or 5 times	218	(25.3)	159	(25.8)		164	(18.9)	134	(21.6)	
Six times or more	126	(14.6)	87	(14.1)		72	(8.3)	50	(8.1)	
Marital status					0.196					0.363
Married	542	(62.8)	415	(67.3)		470	(54.0)	351	(56.6)	
Cohabitating	224	(26.0)	150	(24.3)		260	(29.9)	163	(26.3)	
Divorced, separated, widowed, or never married	97	(11.2)	52	(8.4)		140	(16.1)	106	(17.1)	
Health insurance					0.248					0.124
Yes	510	(59.1)	344	(55.8)		611	(70.2)	407	(65.6)	
No	353	(40.9)	273	(44.2)		259	(29.8)	213	(34.4)	
Age of partner (mean, SD)	29.0	(6.7)	28.6	(6.4)	0.338	27.2	(6.4)	27.2	(6.4)	0.783
Education of partner					0.073					0.210
Did not complete primary	198	(22.9)	144	(23.3)		142	(16.3)	128	(20.6)	
Completed primary	118	(13.7)	94	(15.2)		114	(13.1)	87	(14.0)	
Completed secondary	214	(24.8)	191	(31.0)		243	(27.9)	167	(26.9)	
Above secondary	206	(23.9)	107	(17.3)		218	(25.1)	124	(20.0)	
NA/do not know	127	(14.7)	81	(13.1)		153	(17.6)	114	(18.4)	
Socioeconomic status					0.074					<0.001
Lowest	156	(18.1)	144	(23.3)		188	(21.6)	171	(27.6)	
Lower	155	(18.0)	141	(22.9)		112	(12.9)	132	(21.3)	
Middle	196	(22.7)	104	(16.9)		174	(20.0)	118	(19.0)	
Higher	169	(19.6)	120	(19.4)		192	(22.1)	106	(17.1)	
Highest	187	(21.7)	108	(17.5)		204	(23.4)	93	(15.0)	
Household size (mean, SD)	6.3	(3.1)	6.3	(3.2)	0.783	6.6	(3.7)	6.5	(3.1)	0.681

^†^Rao–Scott cluster-adjusted chi-squared test was used to compare the proportions of a categorical variable by arm. Mixed-effect linear regression models were used to compare the mean of a continuous variable by arm.

NA, not applicable; SD, standard deviation.

Table 5 presents the effectiveness of the interventions based on mixed-effects logistic regression models. The package of CoC interventions improved CoC completion (adjusted odds ratio [AOR] for DiD = 1.77; 95% confidence interval [CI]: 1.08 to 2.92; *p* = 0.024). NNT for the reduction was 12.0. It also reduced maternal complications requiring hospitalization during pregnancy (AOR for DiD = 0.49; 95% CI: 0.29 to 0.83; *p* = 0.008; NNT = 15.4), among other health service and morbidity outcomes. Maternal mortality did not significantly decrease during the trial period (AOR for DiD = 1.60; 95% CI: 0.40 to 6.34; *p* = 0.507) but was found to significantly decrease after the trial period (AOR for DiD = 0.11; 95% CI: 0.01 to 1.00; *p* = 0.050; NNT = 576). Perinatal mortality did not show a significant difference during the trial period (AOR for DiD = 0.81; 95% CI: 0.58 to 1.11; *p* = 0.185) and after the trial period (AOR for DiD = 0.88; 95% CI: 0.60 to 1.30; *p* = 0.520). Likewise, neonatal mortality did not show a significant difference during the trial period (AOR for DiD = 0.97; 95% CI: 0.63 to 1.49; *p* = 0.888) and after the trial period (AOR for DiD = 1.12; 95% CI: 0.62 to 2.01; *p* = 0.713).

**Table 5:** Effectiveness of interventions.

	Effectiveness of interventions (during the trial period)				Effectiveness of interventions (after the trial period)				ICC
	AOR	(95% CI)	*p*-value	NNT	AOR	(95% CI)	*p*-value	NNT	
*Health service and morbidity outcomes*									
Completed CoC	**1.77**	**(1.08 to 2.92)**	**0.024**	**12.0**					0.111
PNC within 48 hours	0.98	(0.63 to 1.50)	0.913						0.178
Maternal complications during pregnancy which required hospitalization	**0.49**	**(0.29 to 0.83)**	**0.008**	**15.4**					0.101
Maternal complications during and immediately after the delivery which required hospitalization	1.28	(0.77 to 2.13)	0.345						0.087
Maternal complications within 6 weeks of delivery which required hospitalization	0.82	(0.28 to 2.41)	0.716						<0.001
Child’s danger signs within 6 weeks of delivery which required hospitalization	0.97	(0.43 to 2.21)	0.945						0.048
*Mortality outcomes*									
Perinatal mortality	0.81	(0.58 to 1.11)	0.185		0.88	(0.60 to 1.30)	0.520		0.011
Neonatal mortality	0.97	(0.63 to 1.49)	0.888		1.12	(0.62 to 2.01)	0.713		0.020
Maternal mortality	1.60	(0.40 to 6.34)	0.507		**0.11**	**(0.01 to 1.00)**	**0.050**	**576**	0.033

Mixed-effects logistic regression models with random intercept at health and demographic surveillance site and community levels were used to estimate AOR. Unstructured variance and covariance matrix was specified.

Health service and morbidity outcomes were analyzed by pooling 2 datasets from the baseline and follow-up surveys and using the model including the following covariates: the woman’s age and education, parity, marital status, health insurance, the partner’s age and education, socioeconomic status (quintile-defined categories), and household size to adjust for differences in the baseline characteristics of women and children. In the model for child’s danger signs within 6 weeks of delivery which required hospitalization, marital status was not included to achieve the convergence in the mixed-effects model.

Mortality outcomes were analyzed by pooling 3 datasets before the study periods, during the study periods, and after the study periods and using the model without other covariates due to unavailability of these covariates in the datasets.

For all the outcomes, the AOR of “effectiveness of interventions” was calculated based on the estimated coefficient of the interaction term between the variables “during (or after) the trial period” and “living in a subdistrict in the intervention arm.” NNT was estimated as cluster adjusted and as the reciprocal of the estimated coefficient of the interaction term based on mixed-effects linear regression model with random intercept at health and demographic surveillance site and community levels.

AOR, adjusted odds ratio; CI, confidence interval; CoC, continuum of care; ICC, intraclass correlation coefficient; NNT, number needed to treat; PNC, postnatal care.

S1 Table presented differences in CoC completion by the characteristics of the nearest health facilities and subdistricts as the results of interaction term analyses. First, woman–child pairs in the control arm were categorized into 2 groups by living closely to an intervention subdistrict or not. Among those living in a control subdistrict, the level of improvement in CoC completion was not significantly different from those living in an intervention subdistrict if they lived close to an intervention subdistrict (AOR for DiD = 0.80; 95% CI: 0.43 to 1.48; *p* = 0.479). If woman–child pairs lived in a control subdistrict and far from an intervention subdistrict, the level of improvement in CoC completion was significantly different from those living in an intervention subdistrict (AOR for DiD = 0.45; 95% CI: 0.26 to 0.78; *p* = 0.004). Second, woman–child pairs in each arm were categorized into 4 groups by the types of the nearest health facility. In the intervention arm, woman–child pairs had a higher level of improvement in CoC completion if their nearest facility was CHPS (AOR for DiD = 2.12; 95% CI: 1.01 to 4.42; *p* = 0.046) compared to those nearest facility was a public hospital. Third, woman–child pairs in each arm were categorized into 3 groups by the existence of higher-tier health facilities in a subdistrict of living. Compared to those living in a control subdistrict with both public hospitals and health centers, woman–child pairs had a higher level of improvement in CoC completion if they lived in an intervention subdistrict without public hospitals or health centers (AOR for DiD = 5.47; 95% CI: 1.87 to 16.0; *p* = 0.002) and with both public hospitals and health centers (AOR for DiD = 11.0; 95% CI: 3.08 to 39.3; *p* < 0.001). Fourth, woman–child pairs in each arm were categorized into 2 groups by the density of health facilities in their subdistrict of living. Compared to those living in a control subdistrict that had a higher density of health facility, woman–child pairs had a higher level of improvement in CoC completion if they lived in an intervention subdistrict with a higher density of health facilities (AOR for DiD = 3.40; 95% CI: 1.75 to 6.59; *p* < 0.001). Fifth, woman–child pairs in each arm were categorized into 2 groups by the dispatch of midwives in their subdistrict of living. Compared to those living in a control subdistrict that had at least 1 facility with a midwife, woman–child pairs had a higher level of improvement in CoC completion if they lived in an intervention subdistrict that had at least 1 facility with a midwife (AOR for DiD = 1.98; 95% CI: 1.23 to 3.21; *p* = 0.005).

S2 Table presents changes in the choice of a health facility for the first ANC, delivery, and the first PNC between the arms and the baseline and follow-up surveys. Among those who were living in a subdistrict in the control arm, the percentage of those who visited a health facility in the intervention arm increased by 7.0 percentage points (from 20.1% to 27.1%) for the first ANC, by 4.8 percentage points (from 26.3% to 31.1%) for delivery, and by 5.3 percentage points (from 16.2% to 21.5%) for the first PNC.

## Discussion

In this cluster-randomized controlled trial, an integrated package of interventions aimed at enhancing CoC in MNCH was found to increase CoC completion and reduced maternal complications requiring hospitalization during pregnancy. It reduced maternal mortality after the trial period but not during the trial period. It did not show evidence of reducing perinatal and neonatal mortality.

The package of interventions substantially increased CoC completion. In the intervention arm, 84.6% of women and children received the package of CoC interventions. Based on our analysis, increased CoC completion was mainly driven by improvements in the coverage of PNC. Specifically, the retention of women and children as inpatients at a health facility (B-1) and PNC by home visit (B-2) contributed to improving the coverage of PNC. The coverage of SBA delivery reached approximately 80% during the trial period. The majority of those who had SBA delivery received PNC within 48 hours. During the trial period, 73% of woman–child pairs in the intervention arm and 71% in the control arm received the first PNC within 48 hours in a health facility. Thus, improvements in the place of delivery and longer retention at a health facility were the main drivers of increasing CoC completion. Home visits are an alternative option of providing PNC, especially when women and children cannot stay longer at a health facility after delivery. In the intervention arm, 14 health facilities among 56 provided B-1 to encourage women and children to stay longer after delivery. The other health facilities did not have sufficient rooms and human resources for B-1. In these health facilities, B-2 was an important option so that women and children could receive PNC at home. Additionally, A-1 and A-2 provided women with education about the importance of and schedule for PNC, which may have contributed to this improvement in PNC coverage.

Supplementary analyses also highlighted strong spillover related to intervention effectiveness. In the control arm, CoC completion differed depending on whether woman–child pairs lived in a subdistrict close to an intervention subdistrict. In addition, more woman–child pairs received MNCH services in a health facility located in an intervention subdistrict at follow-up. Thus, the CoC interventions in this study might affect women’s choices regarding health facilities.

The package of CoC interventions in this study reduced maternal mortality after the trial period and maternal complications requiring hospitalization during pregnancy at follow-up. Maternal mortality can be reduced by the detection and provision of care for women with complications [35–38]. This reduction could be attributed to the effectiveness of the CoC card (A-1) and orientation (A-2) interventions. As shown in the Methods section, the package was implemented at 2 different time points: early pregnancy and after delivery. The CoC orientation was provided so that health workers could understand the importance of CoC and repeatedly disseminate this information to women. The use of the CoC card, in turn, served to provide visualization for services received in all stages; thus, it enabled health workers to explain the importance of CoC to pregnant women. Women could also use the CoC card at home to explain the importance of receiving subsequent ANC, delivering at a health facility, and receiving PNC to their family members and to keep a list the components of birth preparedness [39]. Thus, A-1 and A-2 may contribute to enhancing access to care upon complications, ensuring birth preparedness, and facilitating early decisions regarding the place of delivery. These findings corroborate with previous studies on the association between ANC and improved access to delivery and postpartum services in Asia and Africa [40–42] and on the role of home-based records [18–20,43]. Moreover, these 2 interventions are relatively inexpensive and easily applied. For example, A-2 could be part of a regular orientation conducted by health administrators, and the CoC card cost US$0.50 per woman to print.

Supplementary analyses also showed that the effectiveness of the intervention package might have been influenced by area-specific characteristics of health services (e.g., health facility type and midwife allocation). Indeed, health systems have been considered key elements for improving community health [8,44,45]. Strong health systems endeavor to help women and children access MNCH services regardless of barriers at the individual level. Moreover, these systems ensure that different levels of health facilities remain coordinated, particularly for women and children who receive MNCH services at different health facilities along the CoC. The study area had community-based health planning services and facilities that delivered MNCH services. In Ghana, the CHPS program has been implemented so that women in the rural setting can access health services through the combined efforts of health professionals and the community: In this program, community health officers and nurses work together with the community in their catchment area to enhance the provision of MNCH services, which includes visiting women’s homes [28,46–48]. These roles have been reported as positive for the provision of community-based health services and also been documented in Africa [48–51]. Particularly, home visits by community health workers improve neonatal survival through the early detection of danger signs and the treatment of illnesses [25,26,46].

Nonetheless, the results of this study indicate that perinatal and neonatal mortality rates were not significantly improved by the interventions because both arms showed improvement. In Ghana, although the neonatal mortality rate should be further reduced to achieve the SDGs, it has declined from 36 (in 2000) to 27 (in 2016) among 1,000 live births [21]. In this study, it has expected that the utilized package of interventions would improve early detection of complications and danger signs among women and children. However, studies have shown that a considerable part of perinatal and neonatal mortality can be reduced through strengthening emergency obstetric care [8,38,52]. In Ghana, only 21% of deliveries took place at health facilities with comprehensive emergency obstetric care in 2010 [53]. The scope of this intervention did not include improvements in emergency obstetric care or the quality of MNCH services during ANC, delivery, and PNC. Thus, enhancing access to MNCH services should be made in line with an improvement in emergency obstetric care and the quality of MNCH services.

Although this study presented novel information, it also had the following limitations. First, the type and scale of the health facilities (the units for implementing the interventions) and the size of the subdistricts (the units of cluster randomization) were diverse. The intervention was implemented by making the best use of the existing health systems and using an effectiveness–implementation hybrid design. Furthermore, this study incorporated random effects at the community level to control for unobserved differences in characteristics.

Second, this study found strong contamination for intervention effectiveness. Thus, to examine this contamination, this study conducted separate analyses that considered the differences in health systems and geographic locations at the subdistrict level, in addition to the intention-to-treat analysis (Table 4). Moreover, this contamination may also be attributed to specific characteristics of this study. This study was conducted amid routine health service provision settings, thereby allowing for women living in an area in the control arm to receive MNCH services at a health facility in the intervention arm and vice versa. Additionally, a public hospital in the study area was intentionally allocated to the intervention arm because many women who lived in an area in the control arm visited it for MNCH services. These characteristics were part of the study design because this public hospital played a key leadership role in medical and public health services in the study area; thus, it would have been difficult to implement the interventions without their leadership. Another reason for such contamination could relate to the awareness of the importance of the CoC among health workers, as this information had already been shared among health administrators: In 2013, the annual report of the GHS (Ghana’s government organization for health service provision) addressed the importance of the CoC for MNCH, which was published before the intervention was implemented.

Third, the sample used to assess mortality reduction might not have been sufficient to provide accurate numbers, particularly for maternal mortality, since one of the study sites (Dodowa) was excluded from the mortality assessment. Thus, since the number of maternal mortality cases was small, the hypothesis testing was sensitive to small changes in the number of mortality cases.

## Conclusions

This study found that an integrated package of CoC interventions increased CoC completion and decreased maternal complications requiring hospitalization during pregnancy and maternal mortality after the trial period. These interventions combined the use of inexpensive home-based records (CoC card) and different types of encouragement to provide specific services. These services had already been defined in the national guidelines as important, but had yet to be fully implemented, mainly owing to the resource limitations in Ghana.

This study indicates that the level of CoC completion among women and children can be improved in a real-world setting; however, the number of cases used for the mortality assessment may not have been sufficient to provide conclusive findings. Thus, future studies are warranted to further evaluate the effectiveness of the package over a longer period of monitoring time. This study highlights the importance of improving health systems in ways that can accelerate the effectiveness of the intervention package, particularly by providing more community-based health services and upgrading human resources in the health sector.

## Supporting information

S1 CONSORT Checklist CONSORT 2010 checklist of information to include when reporting a cluster-randomized trial. (DOCX)

S1 Table Characteristics of subdistricts associated with continuum of care completion (*n* = 2,970). CoC, continuum of care.
(DOCX)

S2 Table Choice of a health facility in the intervention or control arm (*n* = 2,970). (DOCX)

S1 Protocol Ghana EMBRACE Implementation Research–Research protocol. (PDF)

## Abbreviations

ANC: antenatal care
AOR: adjusted odds ratio
CHPS: community-based health planning and services
CI: confidence interval
CoC: continuum of care
DiD: difference in differences
GHS: Ghana Health Service
HDSS: Health and Demographic Surveillance System
LMICs: low- and middle-income countries
MNCH: maternal, newborn, and child health
NNT: number needed to treat
PNC: postnatal care
PSU: primary sampling unit
SBA: skilled birth attendant
SDGs: Sustainable Development Goals
