# Supplementary material for: Evaluation of a package of continuum of care interventions for improved maternal, newborn, and child health outcomes and service coverage in Ghana: A cluster-randomized trial
Source: PLoS Med. 2021 Jun 25;18(6):e1003663. doi: 10.1371/journal.pmed.1003663 (PMC8232410; doi:10.1371/journal.pmed.1003663)
Supplement: S1 Table — CoC, continuum of care. (DOCX) [file pmed.1003663.s002.docx]

**S1 Table.** **Characteristics of sub-districts associated with continuum of care completion (n=2,970)**

|  | CoC completion (%) | | Rate ratio (%) (DiD) | DiD estimator | | |
| --- | --- | --- | --- | --- | --- | --- |
| Variable of interest | Base-line | Follow-up |  | AOR | (95% CI) | p-interaction |
| Living in an intervention sub-district^†^ | 7.5 | 47.1 | - | 1 |  |  |
| Living in a control sub-district |  |  |  |  |  |  |
| Close to an intervention sub-district | 10.5 | 54.5 | -17.3 | 0.80 | (0.43 to 1.48) | 0.479 |
| Not close to an intervention sub-district | 8.5 | 31.5 | -41.0 | **0.45** | **(0.26 to 0.78)** | **0.004** |
|  |  |  |  |  |  |  |
| *Nearest health facility characteristics* |  |  |  |  |  |  |
| Type of the nearest facility |  |  |  |  |  |  |
| In an intervention sub-district |  |  |  |  |  |  |
| Public hospital^†^ | 15.8 | 59.8 |  | 1 |  |  |
| Health centre | 6.5 | 49.4 | 100.0 | 1.75 | (0.69 to 4.45) | 0.238 |
| CHPS | 5.4 | 41.6 | 103.4 | **2.12** | **(1.01 to 4.42)** | **0.046** |
| Private facility | 9.2 | 20.8 | -40.6 | 0.46 | (0.13 to 1.59) | 0.219 |
| In a control sub-district |  |  |  |  |  |  |
| Public hospital | 6.2 | 41.1 | 75.8 | 1.13 | (0.36 to 3.62) | 0.832 |
| Health centre | 8.9 | 37.5 | 11.4 | 0.99 | (0.43 to 2.31) | 0.987 |
| CHPS | 8.4 | 43.5 | 37.1 | 1.16 | (0.56 to 2.39) | 0.685 |
| Private facility | 7.5 | 51.5 | 81.4 | 1.51 | (0.50 to 4.57) | 0.470 |
|  |  |  |  |  |  |  |
| Type of interventions at the nearest facility |  |  |  |  |  |  |
| In an intervention sub-district |  |  |  |  |  |  |
| Distribution of CoC card (A-1) and CoC orientation (A-2) only | 12.4 | 44.7 | -37.1 | 0.68 | (0.37, 1.27) | 0.224 |
| A-1, A-2 and 24-hour retention as an inpatient at a health facility (B-1) | 23.7 | 75.5 | -46.8 | 1.22 | (0.41, 3.64) | 0.719 |
| A-1, A-2 and postnatal care by home visit (B-2) | 3.7 | 34.8 | 105.1 | 1.96 | (0.96, 4.02) | 0.064 |
| All interventions | 9.0 | 60.1 | 37.8 | 1.74 | (0.89, 3.42) | 0.112 |
| In a control sub-district^†^ | 8.2 | 41.8 | - | 1 |  |  |
|  |  |  |  |  |  |  |
| *Sub-district characteristics* |  |  |  |  |  |  |
| Distance from a main road |  |  |  |  |  |  |
| In an intervention sub-district |  |  |  |  |  |  |
| Along a main road | 8.5 | 46.3 | -44.2 | 0.62 | (0.16 to 2.37) | 0.488 |
| Far from a main road | 0.9 | 52.7 | 500.1 | 8.57 | (0.77 to 95.28) | 0.080 |
| In a control sub-district |  |  |  |  |  |  |
| Along a main road | 10.2 | 40.9 | -58.9 | 0.40 | (0.10 to 1.53) | 0.182 |
| Far from a main road^†^ | 3.3 | 32.2 | - | 1 |  |  |
|  |  |  |  |  |  |  |
| Existence of higher-tier health facilities |  |  |  |  |  |  |
| In an intervention sub-district |  |  |  |  |  |  |
| With both public hospitals and health centres | 7.0 | 78.0 | 259.6 | **11.0** | **(3.08 to 39.3)** | **<0.001** |
| With either public hospitals or health centres | 8.2 | 40.6 | 59.8 | 1.93 | (0.74 to 5.08) | 0.180 |
| With neither public hospitals nor health centres | 6.6 | 47.2 | 130.8 | **5.47** | **(1.87 to 16.0)** | **0.002** |
| In a control sub-district |  |  |  |  |  |  |
| With both public hospitals and health centres^†^ | 10.1 | 31.3 | - | 1 | - |  |
| With either public hospitals or health centres | 8.1 | 33.3 | 32.7 | 1.89 | (0.67 to 5.30) | 0.228 |
| With neither public hospitals nor health centres | 10.4 | 50.4 | 56.4 | 2.61 | (0.93 to 7.29) | 0.068 |
|  |  |  |  |  |  |  |
| Density of health facility |  |  |  |  |  |  |
| In an intervention sub-district |  |  |  |  |  |  |
| With low density of health facilities per 100 pregnancy cases a year | 7.4 | 41.8 | 57.9 | 1.61 | (0.95 to 2.74) | 0.079 |
| With high density of health facilities per 100 pregnancy cases a year | 7.9 | 58.9 | 108.4 | **3.40** | **(1.75 to 6.59)** | **<0.001** |
| In a control sub-district |  |  |  |  |  |  |
| With low density of health facilities per 100 pregnancy cases a year | 2.9 | 34.4 | 231.6 | **3.64** | **(1.26 to 10.5)** | **0.017** |
| With high density of health facilities per 100 pregnancy cases a year^†^ | 11.6 | 41.5 | - | 1 | - |  |
|  |  |  |  |  |  |  |
| Midwife |  |  |  |  |  |  |
| In an intervention sub-district |  |  |  |  |  |  |
| Without midwife | 2.9 | 12.1 | 2.6 | 0.60 | (0.18 to 1.97) | 0.403 |
| Having at least one facility with midwife | 8.4 | 53.8 | 57.4 | **1.98** | **(1.23 to 3.21)** | **0.005** |
| In a control sub-district |  |  |  |  |  |  |
| Without midwife | 3.3 | 28.9 | 115.2 | 2.06 | (0.55 to 7.76) | 0.287 |
| Having at least one facility with midwife^†^ | 10.2 | 41.5 | - | 1 | - |  |

DiD: difference-in-differences; AOR=adjusted odds ratio; CI=confidence interval.

^†^:Reference category for rate ratio and AOR.

Rate ratio presented above is calculated as follows:

$$\frac{\frac{CoC completion rate at the follow-up in the category of interest}{CoC completion rate at the baseline in the category of interest}}{\frac{CoC completion rate at the follow-up in the reference category}{CoC completion rate at the baseline in the reference category}}.$$

To calculate AOR for DiD estimator, mixed-effects logistic regression models were used with completed CoC as the dependent variable. The dataset pooled the baseline and follow-up dataset. AOR for DiD estimator was computed as the interaction term between time (at the follow-up period) and the variable of interest presented in the row header. The model controlled for the following covariates: the woman’s age and education, parity, marital status, health insurance, the partner’s age and education, socioeconomic status (quintile-defined categories), household size, and time to access the nearest health facility. Main road was defined as a national road (national route, inter-regional route, and major or minor regional routes) in Ghana. The variable “living in a sub-district with low density of health facilities per 100 pregnancy cases a year” had two values (“low” or “high”). It was evaluated if a sub-district had the density of health facilities per 100 pregnancy cases a year lower than the median or not.
