# Supplementary material for: Evaluation of a package of continuum of care interventions for improved maternal, newborn, and child health outcomes and service coverage in Ghana: A cluster-randomized trial
Source: PLoS Med. 2021 Jun 25;18(6):e1003663. doi: 10.1371/journal.pmed.1003663 (PMC8232410; doi:10.1371/journal.pmed.1003663)
Supplement: S2 Table — (DOCX) [file pmed.1003663.s003.docx]

**S2 Table.** **Choice of a health facility in the intervention or control arm (n=2,970)**

|  | Women living in a sub-district in the intervention arm | | | | Women living in a sub-district in the control arm | | | |
| --- | --- | --- | --- | --- | --- | --- | --- | --- |
|  | Baseline (n=863) | | Follow-up (n=870) | | Baseline  (n=617) | | Follow-up  (n=620) | |
|  | n | % | n | % | n | % | n | % |
| *For the first antenatal care* |  |  |  |  |  |  |  |  |
| Intervention facility | 553 | (64.1) | 659 | (75.7) | 124 | (20.1) | 168 | (27.1) |
| Control facility | 84 | (9.7) | 79 | (9.1) | 287 | (46.5) | 311 | (50.2) |
| Out of study area | 113 | (13.1) | 130 | (14.9) | 124 | (20.1) | 140 | (22.6) |
| Did not receive care | 113 | (13.1) | 2 | (0.2) | 82 | (13.3) | 1 | (0.2) |
|  |  |  |  |  |  |  |  |  |
| *For delivery* |  |  |  |  |  |  |  |  |
| Intervention facility | 454 | (52.6) | 516 | (59.3) | 162 | (26.3) | 193 | (31.1) |
| Control facility | 52 | (6.0) | 68 | (7.8) | 155 | (25.1) | 161 | (26.0) |
| Out of study area | 114 | (13.2) | 129 | (14.8) | 135 | (21.9) | 145 | (23.4) |
| Delivered out of a facility | 243 | (28.2) | 157 | (18.0) | 165 | (26.7) | 121 | (19.5) |
|  |  |  |  |  |  |  |  |  |
| *For the first postnatal care* |  |  |  |  |  |  |  |  |
| Intervention facility | 493 | (57.1) | 593 | (68.2) | 100 | (16.2) | 133 | (21.5) |
| Control facility | 57 | (6.6) | 64 | (7.4) | 261 | (42.3) | 268 | (43.2) |
| Out of study area | 210 | (24.3) | 178 | (20.5) | 198 | (32.1) | 194 | (31.3) |
| Did not receive care | 103 | (11.9) | 35 | (4.0) | 58 | (9.4) | 25 | (4.0) |
