# Supplementary material for: Evaluation of a package of continuum of care interventions for improved maternal, newborn, and child health outcomes and service coverage in Ghana: A cluster-randomized trial
Source: PLoS Med. 2021 Jun 25;18(6):e1003663. doi: 10.1371/journal.pmed.1003663 (PMC8232410; doi:10.1371/journal.pmed.1003663)
Supplement: S1 Protocol — (PDF) [file pmed.1003663.s004.pdf]

Ghana EMBRACE
Implementation Research
-Research Protocol-

July 2015

The University of Tokyo, Japan

In collaboration with

Ghana Health Service

Ver. 5

ACRONYMS AND ABBREVIATIONS

ANC	Antenatal Care
CHN	Community Health Nurse
CHO	Community Health Officer
CHPS	Community-Based Health Planning and Services
CHV	Community Health Volunteer
CKI	Community Key Informant
CoC	Continuum of Care
DHMT	District Health Management Team
EMBRACE	Ensure Mothers and Babies Regular Access to Care
FGD	Focus Group Discussion
GHS	Ghana Health Service
HDSS	Health Demographic Surveillance System
HIV	Human Immunodeficiency Virus
HRC	Health Research Center
JICA	Japan International Cooperation Agency
MDG	Millennium Development Goals
MMR	Maternal mortality ration
MNCH	Maternal, neonatal and child health
NMR	Neonatal Mortality Ratio
PNC	Postnatal Care

PMR	Perinatal Mortality Ratio
RHA	Regional Health Administration
SBA	Skilled Birth Attendant
SDHMT	Sub-district Health Management Team
TBA	Traditional Birth Attendant
ToT	Training of Trainers
U5MR	Under-five mortality rate
WHO	World Health Organization

Table of Contents

ACRONYMS AND ABBREVIATIONS 2
RESEARCH FRAMEWORK 6
Study Locations 6
Study Management 6
Data Management 7
Sponsor 7
Funding and Resources 7
INTRODUCTION 8
Background 8
Rationale for Study 12
Hypothesis 12
Objectives 13
METHODS 15
Study Setting 15
Overview of Study Design 16
Trial design 16
Study schedule 17
Outcome measures 17
Study Population 18
The EMBRACE Intervention 19
(A-1) Utilization of maternal, neonatal and child health continuum of care card 21
(A-2) Continuum of care in maternal, neonatal and child health reorientation 23
(B-1) 24-hour retention of mothers and their infants after delivery 24
(B-2)Postnatal care by home visits 25
Equipment and Materials 26
Quality Assurance 27
Preparatory Activities 27
Stakeholder meeting 27
Community leaders meeting 28
Community durbars 29
Randomization 29
Participant withdrawal 30
Impact Evaluation 31

Data management 32
Statistical Analysis 32
Problems Anticipated 33
Ethical Considerations 33
Plan for Dissemination 34
REFERENCES 35
Information Sheet for Women Participants 37
Informed Consent Form for Women Participants 39
Information Sheet for MNCH Care Providers 40
Informed Consent Form for MNCH Care Providers 42
Information Sheet for Women Participants on taking photos and movies 44
Informed Consent Form for Women Participants on taking photos and movies 46
Information Sheet for MNCH Care Providers on taking photos and movies 47
Informed Consent Form for MNCH Care Providers on taking photos and movies 49
Continuum of Care Card .... エラー! ブックマークが定義されていません。
In-depth Interview Guide for Women 50

RESEARCH FRAMEWORK

Study Locations

Institute	Address
Department of Community and Global Health, Graduate School of Medicine, The University of Tokyo	7-3-1, Hongo, Bunkyo-ku, Tokyo, 113-0033 Japan
Dodowa Health and Demographic Surveillance System site	Shai Osu district, Ningo Prampram district, Greater Accra, Ghana
Kintampo Health and Demographic Surveillance System site	Kintampo-North district, Kintampo-South district, Brong-Ahafo, Ghana
Navrongo Health and Demographic Surveillance System site	Kassena-Nankana East district, Kassena-Nankana district, Upper East, Ghana

Study Management

- **Principal investigator**

Masamine Jimba

Department of Community and Global Health, Graduate School of Medicine, The University of Tokyo

7-3-1 Hongo, Bunkyo-ku, Tokyo, Japan

Tel: +81-3-5841-3698 / Fax: +81-3-5841-3422 / E-mail: mjimba@m.u-tokyo.ac.jp

- **Co-Principal investigator**

Abraham Hodgson

Research and Development Division, Ghana Health Service, Ministry of Health

Accra, Ghana

Tel: 233-3021-684271 / Fax: 233-3021-666808 / E-mail: info@ghsmail.org

Data Management

- Department of Community and Global Health, Graduate School of Medicine, The University of Tokyo

  7-3-1 Hongo, Bunkyo-ku, Tokyo, Japan
- Dodowa Health Research Center, Greater Accra, Ghana
- Kintampo Health Research Center, Brong-Ahafo, Ghana
- Navrongo Health Research Center, Upper East, Ghana

Sponsor

Japan International Cooperation Agency (JICA)

1-6th floor, Nibancho Center Building 5-25, Niban-cho, Chiyoda-ku, Tokyo, 102-8012, Japan

Tel: +81-3-5226-8368

Funding and Resources

This study is financed through a grant from JICA.

INTRODUCTION

Background

Maternal and neonatal morbidity and mortality continue to be prominent public health issues, particularly in developing countries. Maternal mortality remains extremely high, especially in Africa, despite a safe motherhood initiative launched about three decades ago. In the same vein, despite reductions in child mortality over the years, about 8 million children died worldwide in 2010. Of these deaths, 40% occurred during the neonatal period in the first month of life,[3] and 98% of these neonatal deaths now take place in developing countries.[3]

Millennium Development Goals (MDGs) 4 and 5 seek to reduce the under-five mortality rate (U5MR) by two-thirds and the maternal mortality ratio (MMR) by three-quarters, between 1990 and 2015. Progress, however, has been minimal and uneven across countries, especially in Sub-Saharan Africa. Unless an annual 7% decline is attained during the period between 2008and 2015, it appears unrealistic for Sub-Saharan Africa to attain MDG 4.[4]

In that context, the "Ensure Mothers and Babies Regular Access to Care" (EMBRACE) initiative was launched in 2010. This initiative was a strategy to step up Japan's concerted efforts to help achieve the health-related MDGs in developing countries, particularly regarding maternal and child health.[5] To extend the benefits of the EMBRACE initiative and evidence-based practice in health policy, the Ghana EMBRACE implementation research project was launched in 2012.Following the results of the formative research, a package of interventions was developed and will be implemented in three Health and

Demographic Surveillance System (HDSS) sites in Ghana.

Maternal, neonatal and child health in Ghana

Under-five mortality decline has been very slow in Ghana. According to the Ghana Demographic and Health Survey (GDHS) 2008, Ghana's neonatal mortality ratio(NMR) was estimated at 30 deaths per 1,000 live births. This represents only a slight decline from the 35 deaths per 1,000 live births reported in the preceding survey in 2003. Moreover, mortality levels in rural areas are consistently higher than those in urban areas. NMRs for urban and rural settings were 30 and 34 per 1,000 live births, respectively.[6]

In the same vein, Ghana's MMR remains remarkably high despite the efforts made by the Government of Ghana and its international development partners. Recent data show that the MMR for Ghana was 350 deaths per 100,000 live births in 2008.[7] Based on sibling history with verbal autopsy, the Ghana Maternal Health Survey (GMHS) also estimated the pregnancy-related mortality ratio (PRMR) at 580 deaths per 100,000 live births when counting the household deaths in 2007.[8] The major single cause of maternal death was hemorrhage, which yet remains still remains a leading cause (22.9%), followed by abortion (12.3%) and hypertensive disorders of pregnancy (10.4%),[8]In Ghana, the percentage of births attended by a skilled health service provider has registered a similarly slow progress, increasing from 40% in 1988 to only 59% in 2008.[9]

Many of these deaths are preventable, if only appropriate measures could be put into place in advance. In order to support countries in Sub-Saharan Africa to move towards the attainment of the MDGs, the African Regional Reproductive Health Taskforce meeting was held in Dakar in 2003.Consequently, the MDG Acceleration Framework was developed by the Ministry of Health (MoH) and Ghana Health Service (GHS) in

collaboration with development partners in Ghana. The Framework aims to redouble efforts to overcome bottlenecks in implementing interventions that have been proven effective in reducing MMR in Ghana.

Continuum of care in maternal, neonatal and child health

The continuum of care (CoC) in maternal, neonatal and child health (MNCH) has been played up as a strength in improving the health of mother and child.[10] The WHO and other leading global health agencies in particular, have been advocating over the last decade, the potential power of the CoCin the context of MNCH. The concept of CoC has emerged as a new paradigm to address maternal, newborn and child mortality and is crucial to battling all of these forms of mortality.[1112]

However, the definition of CoC has not been clearly established yet, though several attempts have been made.[13-16] The term itself is well known in the health systems are in the context of palliative care nursing.[1013] In the public health field, the concept of CoC has been used in care of people living with HIV/AIDS,[10] to link diagnosis, initiation of care, retention in therapy, and maintenance of a low viral load. With regards to the area of MNCH, the broad consensus on CoC incorporates integrated service delivery from pre-pregnancy to postnatal care, at home, community, and in health facilities. It is often explained by the dimensions of time and space: In the time dimension, CoC spans the period from pre-pregnancy to childhood for women and children. In the space dimension, meanwhile, it reaches from the home, to the community level, and through health facilities.[16]

The components of CoC forming the intervention package to be implemented EMBRACE study are as follows:

(1) At least four antenatal care (ANC) visits with MNCH skilled health service providers at the health facility, within the community, or at home;
(2) Delivery assisted by skilled birth attendants (SBAs);
(3) A minimum of three postnatal care (PNC) visits with MNCH health service providers at a health facility, in the community, or at home within 48 hours, on the seventh day, and at the end of six weeks postpartum consecutively.

The CoC in MNCH provides a framework whereby several evidence-based interventions can be combined and delivered in packages.[13] Interventions included in such packages can be selected out of more than 190 separate interventions, and they can be delivered using this mode of integrated service delivery.[13] As maternal and newborn health are closely related, the main focus of the CoC was once on maximizing maternal and newborn survival through promoting regular access to reproductive and maternal health care for women, particularly during pregnancy and childbirth.[14] However, the CoC also can be effective to improve care and to reduce morbidity, too. An effective intervention is required to cover the cracks along with the time and space dimensions of the CoC by strengthening the linkages between various levels of health systems and between service delivery packages.

Implementation research

To make health service interventions more effective, it is necessary to understand how interventions work in “real world” setting.[17] Implementation research has a role in solving such implementation problems, taking into account the social, demographic, cultural, and economic environment; the epidemiological conditions; and the institutional setting. Both qualitative and quantitative research methods can be used in implementation research,

and several approaches have been developed to deal with the associated questions: pragmatic trials, effectiveness implementation hybrid trials, quality improvement studies, participatory action research, and mixed methods.[17] These approaches may be applied according to the circumstances prevailing in the intervention areas, with due consideration given to their limitations.

Rationale for Study

According to currently available evidence, Ghana is not on track to achieve MDGs 4 and 5. Despite increases in ANC attendance at the health facilities, and in the number of deliveries attended by skilled health service providers, the needed declines in neonatal and maternal mortality have not been seen. In addition, according to our formative research in Ghana, the CoC completion rate was only 10% among women in Ghana. In particular, an abrupt drop was identified in the care uptake rate at the within-48-hours-postpartum PNC stage, underlining a prominent crack in the CoC time dimension that requires urgent action for improvement.

Ghana has multiple localities, characterized by the unique environmental qualities of each area. Maternal and neonatal deaths are a function of a complex interaction of economic, financial, social, cultural and clinical factors. The diversity of local characteristics demands flexibility in health service provision. As such, it is especially important to conduct the implementation trials in Ghana in the different local contexts.

Hypothesis

Implementation of an integrated package of CoC intervention improves the following

MNCH intervention and implementation impact outcomes within one year among the target population in rural Ghana.

Objectives

The study aims to improve MNCH status by strengthening the MNCH CoC in rural Ghana. Specific objectives are proposed for intervention and implementation phases. In the public health context, a health program is planned toward achieving population health goals or around objectives designed to improve quality of life. An intervention is one component of such a program and a method of initiating a process to attain such goals and objectives. Typically, a program consists of multiple interventions which are planned based on the people's needs and in light of the desired outcome and its impact. However, to integrate the study findings and products into routine and sustainable use, it is necessary to adopt an appropriate concept and theory on which to base the interventions. An implementation process thus comprises the effort of applying the theoretical framework of an intervention to the real circumstances on the ground.[18]

Intervention objectives

- To evaluate the impact of interventions on the CoC completion rate.
- To evaluate the impact of an improved CoC completion rate on health outcomes among mothers and infants.

Implementation objective

- To examine the feasibility of the planned CoC interventions in rural Ghana.

METHODS

Study Setting

The study will be carried out at three different Health and Demographic Surveillance System (HDSS) sites of the GHS in Ghana: Dodowa (Greater Accra region), Kintampo (Brong-Ahafo region), and Navrongo (Upper East region). See figure 1. These three sites were selected based on the high reliability of HDSS data for evaluating the study's impact. The feasibility of implementation will be evaluated in different contexts. The HDSS involves a semi-annual recording of vital demographic events, including pregnancies, births, deaths, and migration, occurring among residents of all households in the HDSS area. Other data are also collected and updated regularly, such as household characteristics, morbidity, and vaccination uptake.

The Dodowa HDSS site registered a surveillance population of approximately 115,000 as of 2011. The site is located only about 40 kilometers away from Accra.[19] Consequently, pregnant women of this site often prefer to access health facilities in Accra to give birth. Delivery of services is, however, generally challenging due to the large land size, covering about 40.5% of the Greater Accra Region. Most of the communities are scattered, with small population sizes.[19]

In Kintampo, the surveillance population numbered approximately 200,000 in 2011.The Kintampo HDSS site is a multi-ethnic area, and farming is the most important economic activity. Apart from the central area, few villages have electricity and most are reached by dirt roads. Access to health facilities is particularly challenging in Kintampo.[19] One-third of pregnant women did not deliver in health facilities, according to our situation analysis.

In Navrongo, the surveillance population numbered approximately 153,000 in 2011.Navrongo is the first area where the Community-based Health Planning and Services (CHPS) program was launched in Ghana. In that context, Community Health Officers (CHOs)contribute to improving the health status of the rural community. Almost 90% of women delivered at health facilities and received PNC within 48 hours of giving birth, according to our situation analysis.

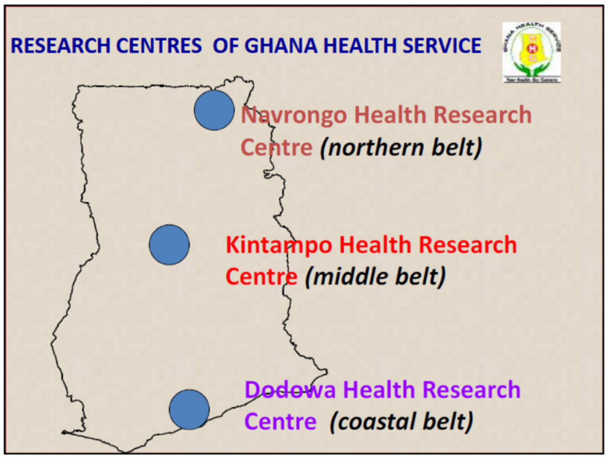

Figure 1 Map of Health Research Centers

Overview of Study Design

Trial design

This study has been designed as an effectiveness-implementation hybrid trial,[17][20][21]testing the effectiveness of the intervention as well as the feasibility of its implementation strategy.[20] Under this study design, the effectiveness will be assessed for both an intervention package and its implementation process by using cluster-randomized allocation to divide participants into intervention and control arms. The study would be conducted in three stages, namely: the baseline, implementation and evaluation phases. Feasibility of the trials will be also be evaluated through monitoring on-going trials by

district health management team (DHMT)/sub-district health management team (SDHMT) supervisors. Supervisors such as the public health nurse, the disease control officer, for e.g. from the DHMT and SDHMT already have monitoring responsibilities, which will be further enforced by the refresher training as part of the intervention.

Study schedule

(i) Baseline survey: 1 July 2014 to 31 July 2014

(ii) Intervention: 1 October 2014 to 31 December 2015

(iii) Evaluation survey: 1 September 2015 to 31 October 2015

(iv) Analysis/writing: From November 2015

Outcome measures

Implementation of an integrated CoC intervention package is expected to improve the following MNCH intervention and implementation impact outcomes within one year within the target population in rural Ghana:

Intervention impact outcomes

The primary outcome of the intervention is 1) CoC completion rate of mothers and infants. Secondarily, outcomes such as 2) the PNC rate within 48 hours postpartum, 3) the rate of complications requiring hospitalization of mothers and their infants for more than 24 hours, and 4) the perinatal mortality ratio (PMR) will be measured. All the data will be compared between intervention and control arms.

Implementation impact outcomes

The implementation impact outcomes are 1) acceptability of CoC card utilization and 2) feasibility of improved PNC rates within 48 hours postpartum by mothers' retention at the health facility or by home visits. These outcomes are to be compared before and after the intervention.

Study Population

Pregnant women will be recruited as participants of the study from the areas of Dodowa, Kintampo and Navrongo HDSS. Eligibility criteria will be as follows.

(i) Participants of the baseline survey:

Study population would be females who got pregnant in the target HDSS sites. The inclusion criteria are those who gave birth within the two years preceding the baseline survey, including live-births and still-births. The exclusion criteria will be those who have infants younger than six weeks of age at the beginning of the data collection.

(ii) Participants of the intervention and evaluation survey:

The study population would be females who got pregnant in the target HDSS sites. The inclusion criteria will be those who are between 16 weeks gestation and six weeks postpartum during the intervention period of A-1, B-1 and B-2 **(1st October 2014 to 31st December 2015)** including live-births and still-births. The exclusion criteria will be those who have infants younger than six weeks of age at the beginning of the data collection**.** From preliminary information gathered from the participating three HDSS sites, during the intervention period, about 2**7,500** mothers are expected to experience pregnancy, delivery, or the postnatal period in the study sites, and these will form the target population in this study. **An**

estimated total of 10,000 mothers are expected to be fully followed up from their first ANC visit to their PNC visit at six weeks post-partum (approximately 5,000 intervention and 5,000 control arms), which accounts for 36 % of all target mothers. The remaining 64% will be also followed up as they will partially receive the intervention; those who registered at their first ANC before the start of intervention, and those whose third PNC period occurs after the intervention period. See table below.

Year	2014										2015											
Month	3	4	5	6	7	8	9	10	11	12	1	2	3	4	5	6	7	8	9	10	11	12
1: Partial follow-up	8,750 mothers																					
2: Full follow-up								10,000 mothers														
3: Partial follow up																8,750 mothers						

Figure 2 Three sub-groups of the target mothers categorized by month of conception

The EMBRACE Intervention

The EMBRACE package of interventions was developed based on formative research surveys conducted in 2012 and 2013. These surveys consisted of situation analyses, assessments of CHOs' working conditions, and analyses of the social capital in the local population. These were elaborated and carried out through close collaboration with the researchers of GHS at the:Dodowa, Kintampo, Navrongo Health Research Centers (HRC) and The University of Tokyo.

The situational analysis survey was conducted to identify the cracks in, barriers to, and

promoters of service reception in continuous MNCH care. In this survey, 1,500 women were recruited from three HDSS sites, all of whom had become pregnant within the one year preceding the survey. They were interviewed using a semi-structured questionnaire. The survey also involved qualitative study components such as key informant interviews and focus group discussions (FGDs). The key informant interviews targeted doctors, midwives, CHOs, and district officers who worked in MNCH. The FGDs targeted mothers who had become pregnant within the last one year, their husbands, and their mothers-in-laws.

Although delivery and laboratory services are provided only at the secondary and tertiary level health facilities, basic CoC services are provided even at the primary level in Ghana. ANC services include general check-ups for mothers, urine testing, hemoglobin testing, prevention of mother-to-child transmission (PMTCT) of HIV, nutritional support, tetanus immunization, and health education for birth preparedness and maternal complications. Delivery services, meanwhile, include skilled delivery, facility referral, and emergency obstetric care. Finally, PNC services include general check-ups for mothers and infants, body weight measurements, infant vaccination, hemoglobin testing, nutritional support, health education for breastfeeding and infant care, and family planning counseling. ANC and PNC services are also provided through home visits.

However, in actual practice, not all of these services are taken up consistently by mothers and infants. CoC as a concept is quite new in Ghana, and the linkages between MNCH services are weak. Consequently, most Ghanaian mothers are not conscious of the availability and importance of continuous MNCH services. In particular, rates of uptake for PNC within 48 hours postpartum have been especially low, likely due to the difficulty faced by mothers in accessing a health facility after home delivery. Moreover, assuming

the mother has no particular health issue, mother and infant are usually discharged within less than 24 hours postpartum.

In light of this context, the following four interventions were developed: utilization of an MNCH CoC card (A-1), CoC reorientation (A-2), 24-hour retention of mother and neonate post-delivery at the health facility (B-1), and PNC provided by home visit within 48 hours after delivery (B-2).Interventions A-1 and A-2will be uniformly implemented in the intervention arm, while B-1 and B-2 will be discretionary according to the sites' resource. In the health facilities of the control arm, conventional MNCH services will be provided. Details of each intervention are explained in the following sections.

(A-1) Utilization of maternal, neonatal and child health continuum of care card

An MNCH CoC card for mothers will be developed to meet four objectives: 1) to encourage mothers to comply consistently with the full maternal and infant care regimen, 2) to educate mothers on the timing and schedule of the necessary MNCH services and visits, 3) to evaluate and monitor mothers' status in terms of care uptake and health-related danger signs, and 4) to establish a strong rapport between mothers and health workers. The card has been created by the Ghana EMBRACE implementation research project team based on the GHS and WHO guidelines. It was reviewed by senior GHS advisors in charge of health education, and approved by the EMBRACE Advisory Board members in February 2014.

The implementation period of this intervention is from September 2014 to **December 2015**. The card will be provided through health service providers to all women who receive ANC, delivery attendance, and PNC services via SBAs such as obstetricians, midwives, or CHOs under the supervision of a midwife. Each woman will receive a card at the timing of her first visit for registration with health facilities or any of the above services. Health service providers include midwives, CHOs, Community Health Nurses (CHNs), and doctors in public, private and faith-based health facilities. These cadres will provide routine MNCH care at the health facility level, at the community level, or at home. Each time a pregnant woman receives CoC services at a health facility, by outreach, or by home visit, the health service providers will put a sticker on the card to show that the mother has received CoC services. The timings at which women receive these stickers include each of the four ANC sessions, with SBA-assisted delivery, and the three PNC sessions (within 48 hours, on the seventh day, and at the end of six weeks postpartum).

Table 1: MNCH CoC card contents

Contents	Main components
**CoC services**	-ANC (4x)<br>-Birth by skilled attendant<br>-PNC (3x)
Essential services	-Hemoglobin test<br>-Malaria drug (IPT)<br>-Tetanus toxoid injections
**Health education**	**-Items for delivery and baby**<br>-Caretaker<br>-Transport<br>-Breastfeeding counseling<br>-Family planning counseling
Danger signs	-Identification of danger signs and associated dates

Through the intervention, the women will also be educated by health workers on birth preparedness practices during ANC visits and about exclusive breastfeeding and family planning during PNC. Upon confirming that the women received these health education messages, the health workers will affix the appropriate seals on their cards. The cards will also contain other relevant information such as blood test results for hemoglobin

assessment, intermittent preventive treatment (IPT) for malaria, tetanus toxoid immunization, and danger sign records. This information is intended to help health workers to see at a glance the women's stages of care and danger signs history, with more detailed information accessible for review in the maternal health record book. The card will be stored in the mothers' handbook and kept with them.

(A-2) Continuum of care in maternal, neonatal and child health reorientation

The CoC in MNCH reorientation will be carried out with health administrators (supervisors of the District Health Management Team (DHMT) and Sub-district Health Team (SDHMT) and health service providers. Its purpose is to reorient supervisors and health service providers toward a solid understanding of the CoC concept in MNCH, with which they can then educate the targeted women on the importance of the CoC. The reorientation will be conducted in August 2014.

The reorientation will be implemented in two stages: training of trainers (ToT), followed by reorientation of the health service providers. The reorientation is mainly focused on introducing CoC concepts in MNCH, the importance thereof, and uses of the CoC promoting card. However, instruction will also be provided on how to conduct interventions and to supervise the DHMT and SDHMT. Each phase of instruction will take three consecutive days and be held in the local DHMT office. Training materials such as manuals and guides were developed by the Ghana EMBRACE implementation research project team during the workshop held in February 2014. All materials were created based on the various guidelines validated by GHS, WHO, and UNICEF, and approved by the EMBRACE Advisory Board members.

In the ToT phase, a master training team will be formed by the Ghana EMBRACE

researchers and regional health administration (RHA) members. This team will deliver the ToT about reorientation to DHMT/SHMT members. In each session, all DHMT/SHMT staff members with responsibility for health facility supervision will attend. The EMBRACE research members from HRCs will also attend as observers.

In the second phase of the training, RHA/DHMT/SHMT staff members who received ToT will, in turn, provide the reorientation to health service providers. Participants in this health service provider reorientation will include MNCH service providers (midwives, CHOs, CHNs, doctors, and health assistants) who work in public, private and faith- based health facilities within the study's intervention arm. Health service provider reorientation sessions will be led by RHA/DHMT/SHMT trainers and HRC researchers who attended the relevant ToT sessions.

The trained DHMT/SDHT staff members will supervise the trained health service providers through a regular monthly supervision in their catchment areas. The aim is to visit each health facility at least once a month to directly observe health providers' service provision. A standard sheet will be developed to support supervision, with space in which to record activities and issues raised.

(B-1) 24-hour retention of mothers and their infants after delivery

Upon delivering, women will be encouraged to stay at the health facilities for at least 24 hours with their neonates to receive PNC. The aim of this intervention is to improve the within-48-hours-postpartum PNC uptake rate of mothers and neonates. The implementation period will run from **October 2014 to December 2015**. This intervention will be conducted only in those health facilities providing delivery service by SBAs and having space for abed for postpartum rest. Approximately 5,850 women are estimated to

use this service during the intervention period. For those who are not fully followed from the first ANC to the PNC at six weeks postpartum, the impact of CoC completion will be analyzed according to the care stages they received during the intervention period. Such data may be analyzed separately from fully followed-up mothers. Intervention impacts in terms of postpartum complications will also be analyzed.

In this intervention, mothers and babies will receive necessary care in the health facility for 24 hours after delivery. During their stay, mothers will be provided nutritious food drink (Milo) offered by the Ghana EMBRACE implementation research project. After ensuring all of the necessary health check points, health service providers will discharge the mothers. In cases where mothers and/or neonates have particular symptomatic complications, midwives will provide supplementary extended care or refer to the district hospital, as appropriate. The women will be advised to come to the health facilities with their neonates for PNC on the seventh day and at the end of six weeks following delivery. If the women do not return to the health facility for PNC as instructed, the health workers will visit their homes to provide PNC service, as part of their routine follow-up duties.

(B-2)Postnatal care by home visits

Functional CHOs will be encouraged to visit mothers and babies for PNC within 48 hours postpartum. Similar to the B-1 intervention, the aim of this intervention is to improve the witin-48-hours-postpartum PNC rate for mothers and neonates. The implementation period will run from **October 2014 to December 2015.** The intervention is composed of two steps. The first step is delivery notification: in case of home delivery, Community Health Volunteers (CHVs), Community Key Informants (CKIs) or Traditional Birth Attendants (TBAs) will inform the CHOs about the labor or delivery. In the case of facility

delivery, when a woman has left the facility before the first PNC, SBAs will convey that information to CHOs so that they can visit the woman at home for PNC. The second step is PNC by home visits: CHOs will visit mothers and their neonates for PNC within 48 hours of delivery for women who delivered at home. If a woman delivers at a health facility but does not receive PNC within 48 hours, CHOs will visit her according to the information obtained from the health facilities. Approximately 8,750 women are estimated to be using this service.

The PNC by home visits will be conducted by functional CHPS zones with compounds. Targeted CHPS coverage is 50% of the total area. The estimated number of CHPS zones is 17 in Dodowa, 18 in Kintampo, and 21 in Navrongo. Delivery information will be communicated to the functional CHPS compounds via CHVs, CKIs, and TBAs. These messengers will receive two Ghana cedis (approximately 1 USD) per labor or delivery message conveyed as part of their communication fee. The money will be paid from the Ghana EMBRACE Implementation Research project budget. The women will be advised to visit health facilities for PNC on the seventh day after delivery. If the women do not come to the health facility, the CHOs will visit their homes to provide PNC service, as part of their routine follow-up duties.

Equipment and Materials

Eligible health facilities will be provided abed for postpartum rest or a motorbike if necessary, as well as a set of materials that aim to assure the minimum stable implementation of the intervention. Before initiating the interventions, the study team will conduct a facility assessment for all health facilities in the study sites and investigate

available equipment and materials. If functional equipment and materials are not available, functional ones will be duly provided. Where necessary, abed for postpartum rest, rechargeable lamp/solar lantern, and torchlight will be provided for all eligible health facilities as part of the B-1 intervention. A motorbike will be provided for health facilities as part of the B-2 intervention. A set of care materials will also be provided for both eligible categories of health facility: blood pressure apparatus for mother and infant, stethoscopes, thermometers, and pen lights.

Quality Assurance

In each HRC, a monitoring team will be formed. This team will be responsible for monitoring all implementation activities of health service providers and for supervision of DHMT/SDHT supervisors. They may join the DHMT/SDHT supervision and directly observe health providers' service provision and supervisors' supportive activities as well. During the initial period of the intervention (for the first four months), monthly monitoring will be undertaken, after which the frequency will decrease gradually.

Preparatory Activities

Several preparatory activities will be organized to help the implementation proceed smoothly.

Stakeholder meeting

The HRCs will organize stakeholder meetings before and after the intervention. The

purpose of the stakeholder meetings is to gain support and credibility for the intervention by eliciting the stakeholders' and the wider community's involvement and ownership in the initiative. In this meeting, the HRCs will invite RHA, DHMT, and SDHMT members from both intervention and control areas. At far as content, the Ghana EMBRACE researchers of the HRCs will present the outline of the Ghana EMBRACE implementation research project, explain the purpose of the proposed package of interventions, and discuss its implications for the health facilities. This includes a detailed discussion to harmonize messages between the research team and stakeholders, as well as feedback on findings from the formative research survey. A list of issues which may arise during the intervention period will be made at the stakeholder meeting and shared with other HRCs.

Community leaders meeting

The HRCs will also organize meetings for community chiefs and key members of the communities in the intervention arm before and after the intervention. The Ghana EMBRACE researchers of the HRCs and DHMT/SDHMT staff members will visit all intervention arm communities during June and July 2014 and hold meetings with the chiefs and key members of the communities. These meeting will aim to obtain the consent of principal community leaders to carry out the interventions in their communities. Each meeting will last one to two hours and take the form of a presentation and discussion. The questions raised during the meetings will be noted and brought to attention of the Ghana EMBRACE research team to feed into the implementation training. When the interventions have been completed in **January 2016,** a second round of community

leaders meetings will be held and the results of the intervention will be shared with the chiefs and key members of the communities.

Community durbars

Community-wide meetings will be held by HRCs and DHMT/SDHMT members in each community of the intervention arm before and after the intervention. The purpose of these meetings will be to introduce the Ghana EMBRACE implementation research project and the intervention contents, and to discuss the importance of community support for its success. Such meetings will be chaired by the community chiefs, inviting the community residents including CHVs, CKIs and TBAs to attend. Once the intervention is completed, in **January 2016,** the second community durbar will be held and the results of the intervention shared with the community.

Randomization

In each HDSS site, sub-districts will be allocated to either the intervention or the control arm. Only Jema and Dumso sub-districts (which used to be a part of Jema) in the Kintampo site will be combined to form a cluster in order to match the Kintampo sub-district with respect to population and number of deliveries. In a total of three HDSS sites, 32 clusters will be set as the Ghana EMBRACE implementation research zone. From each HDSS site, half of the clusters will be allocated as the intervention arm and the other half as the control arm. Cluster stratification will be carried out using restricted stratified randomization to ensure balanced numbers of population, deliveries in each cluster, and

midwives available. Then, 16 pairs of clusters will be matched, with one of each pair being allocated randomly to either the intervention or the control arm. The random sequence of allocation will be computer-generated. Cluster randomization is preferred over individual-level randomization to minimize contamination and for pragmatic purposes with a view to the future scale-up of the intervention. Randomization will be done by a data analyst who is not a primary member of the study team.

Intervention arm

The intervention will be implemented in 16 clusters: four clusters in Dodowa, six clusters in Kintampo, and six clusters in Navrongo. Recipients of the intervention will comprise pairs of woman and their infants who have received maternal and infant services, in addition to the routine MNCH services currently available.

Control arm

The control arm will include 16 clusters: four clusters in Dodowa, six clusters in Kintampo, and six clusters in Navrongo. In the control arm, all women and infants will continue to benefit from the routine MNCH services currently available: ANC, PNC, access to free SBA-attended delivery, and routine interactions with CHOs concerning home-visit and MNCH care.

Participant withdrawal

Participants will be withdrawn from the study if they:

- experience a serious or intolerable adverse event;

- develop or disclose, during the course of the study, symptoms or conditions listed in the exclusion criteria; or
- require early discontinuation for any other reason.

Impact Evaluation

To evaluate the impact of the intervention package, the baseline and evaluation surveys will be conducted in three HDSS sites. The period of the baseline survey will be from July to August 2014. **The intervention period will run from October 2014 to December 2015. Period of the last three months (October to December 2015) will be added to mitigate the decline in the service quality due to the abrupt intervention cessation. The evaluation survey period will be from October to November 2015, and the survey's target will not include the mothers who would newly enter this period, thus the survey results would not be influenced by the addition of the period.**

Individual mother- and infant-level data will be collected regarding their CoC MNCH completion rates, complication rates, within-48-hours-postpartum PNC rates, mortality and adverse outcomes. In the baseline survey, data will be collected through household surveys of mothers who delivered within the two years preceding the survey. In the evaluation survey, mothers will be included if they have received any aspect of MNCH care, even in part, during the intervention period. However, for data from those who cannot be fully followed up during the intervention period, analyses will be performed according to the care stages they received during the intervention period. This may be analyzed separately from mothers who have passed the period from the first ANC session to the PNC at six weeks during the intervention period. Individual mother- and infant-level data will also be obtained from the HDSS data set regarding pregnancy outcomes

and morbidity.

To evaluate the impact of the implementation, a set of data and supporting information will be collected during the supervision by the DHMT/SDHMT. **This includes the status of CoC card distribution and actual users, as well as uptake of PNC within 48 hours postpartum, either by retention of mothers at the facilities or by home visits. In addition, qualitative data of women and health workers will be collected during the monitoring by the researchers. Those include transcript data of interview, audio-visual data (photographic and motion data) which will be collected through the in-depth interviews. Target of in-depth interview will be women and health workers who will be involved in the intervention or control arm.**

Facility-level data including availability of infrastructure, equipment, and MNCH service providers will be collected from each health facility. These data items will be used as covariates that might influence an outcome in the impact evaluation.

Data management

Data collected during visits would be checked for accuracy and completion by supervisors. Data would be double entered into computer using Visual Foxpro software. Verification checks will be done to correct the discrepancies in records.

Statistical Analysis

Baseline and evaluation survey data will be analyzed to assess changes in each outcome in both intervention and control arms. To minimize overestimation of the intervention effect, all intervention impact outcomes will be estimated by intention-to-treat analysis, by which individuals are analyzed according to an allocated arm as the outcome data are

available, regardless of the place where they received the care. Also, all eligible individuals are included in the analysis, whether or not they provided outcome data[22]. Data collected at the individual level will be analyzed using generalized estimating equations (GEE) and mixed effects models for both continuous and binary outcomes[22]. Adjustment factors include items such as basic demographic characteristics and socio-economic characteristics, as well as the characteristics of facilities located in the area where the mother and infant live. Data analysis will be done primarily using Stata version 12.1. (StataCorp LP, College Station, Texas, USA).

Problems Anticipated

Participants' name will not be recorded, so no danger of potential privacy violations will be presented.

Ethical Considerations

Ethical approval will be obtained from the Ethics Review Committee of the GHS, the Institutional Review Boards of Dodowa HRC, Kintampo HRC, and Navrongo HRC in Ghana, as well as the University of Tokyo in Japan. The implementation and research periods are estimated to span five years. Informed consent will be sought from all study participants and health workers before their inclusion in the study. **Especially for photographic and motion data, we will obtain consent on potential exposure of the data at public place, such as at national and international conference.** Permission will be sought from the local health authorities as well as from community leaders.

Study records will be identified by means of study IDs. Participants will be given the option to withdraw at any time during the study with the assurance that this will not affect

their normal service delivery at any of the health facilities.

Plan for Dissemination

The results of the study will first be presented to community members and their leaders at HDSS sites. In addition, policy briefs will be developed in collaboration with the Policy, Planning. Monitoring and Evaluation Division of the GHS and submitted to the Office of the Director-General of GHS and to the Family Health Division. Presentations will be made at the GHS Directors meeting, the Senior Managers Meeting (SMM), as well as relevant international conferences. Papers based on the results will also be submitted for publication in peer-reviewed journals.

REFERENCES

1. WHO. Revised 1990 estimates of maternal mortality: a new approach by WHO and UNICEF. Geneva, Switzerland, 1996.

2. Ngom P, Akweongo P, Adongo P, et al. Maternal mortality among the Kassena-Nankana of northern Ghana. Stud Fam Plann 1999;**30**(2):142-7.

3. WHO. Making a difference in countries: strategic approach to improving maternal and newborn survival and health: ensuring skilled care for every birth. Geneva, Switzerland, 2006:29.

4. Kinney MV, Kerber KJ, Black RE, et al. Sub-Saharan Africa's mothers, newborns, and children: where and why do they die? PLoS Med 2010;**7**(6):e1000294.

5. Ministry of Foreign Affairs Japan. Japan's global health policy: 2011-2015. Tokyo, Japan, 2010.

6. Ghana Statistical Service (GSS) GHSG, ICF Macro. Ghana Demographic and Health Survey 2008, 2009.

7. WHO. World Health Statistics 2011. Geneva, Switzerland, 2011.

8. Ghana Statistical Service (GSS) GHSG, Macro International. Ghana Maternal Health Survey 2007, 2009.

9. UNDP. Beyond the midpoint: achieving the Millennium Development Goals. New York, USA, 2010.

10. McBryde-Foster M, Allen T. The continuum of care: a concept development study. J Adv Nurs 2005;**50**(6):624-32.

11. Martines J, Paul VK, Bhutta ZA, et al. Neonatal survival: a call for action. Lancet 2005;**365**(9465):1189-97.

12. Lawn JE, Tinker A, Munjanja SP, et al. Where is maternal and child health now?

Lancet 2006;**368**(9546):1474-7.

13. Kerber KJ, de Graft-Johnson JE, Bhutta ZA, et al. Continuum of care for maternal, newborn, and child health: from slogan to service delivery. Lancet 2007;**370**(9595):1358-69.

14. The Partnership for Maternal NCH. Strategic Framework 2012 to 2015. 2011.

15. Tinker A, ten Hoope-Bender P, Azfar S, et al. A continuum of care to save newborn lives. Lancet 2005;**365**(9462):822-5.

16. WHO. *The World Health Report 2005: Make Every Mother and Child Count*. Geneva, Switzerland: World Health Organization, 2005.

17. David HP, Taghreed A, Olakunle A, et al. Implementation research: what it is and how to do it. BMJ 2013;347.

18. Brownson RC CG, Proctor EK. *Dissemination and implementation research in health :translating science to practice*. Oxford, UK: Oxford university press, 2012.

19. INDEPTH. INDEPTH Network. Secondary INDEPTH Network. http://www.indepth-network.org.

20. Curran GM, Bauer M, Mittman B, et al. Effectiveness-implementation hybrid designs: combining elements of clinical effectiveness and implementation research to enhance public health impact. Med Care 2012;**50**(3):217-26.

21. Zwarenstein M, Treweek S, Gagnier JJ, et al. Improving the reporting of pragmatic trials: an extension of the CONSORT statement. BMJ 2008;**337**:a2390.

22. Eldridge S, Kerry S. *A practical guide to cluster randomised trials in health services research*. New York, USA: Wiley, 2012.

Information Sheet for Women Participants

Study Title:

EMBRACE Implementation Research in Ghana

Principal Investigator:

- MasamineJimba(Department of Community and Global Health, Graduate School of Medicine, The University of Tokyo)
- Abraham Hodgson (Research & Development Division /Ghana Health Service/Ministry of Health)

Dear Participants,

This study aims to improve maternal, neonatal, and child health status by strengthening the Continuum of Care (CoC) in rural Ghana. In this study, we would like to conduct a questionnaire survey. We won't ask your name in this study. So, your identity will not be disclosed. If you agree to proceed with the study, we will conduct the interview.

This study is approved by the Ethics Committee of the University of Tokyo, the Ghana Health Service (GHS) Ethics Committee, as well as the Ethics Committees of the Kintampo, Dodowa, and Navrongo Health Research Centers. Some of the questions may prove embarrassing for you. However, your participation in this study is entirely voluntary and you may refuse to answer any question if you choose at any time without penalty. The interview will take about **30** minutes. All the information we obtain from you will remain strictly confidential and your responses will never be identified. For the focus group discussions and the in-depth interview, participants' discussions will be recorded for data analysis, but will be totally deleted after analysis is completed.

You may ask any question about the study at this time, and if you have further questions about this study, please do not hesitate to utilize the below contacts.

MasamineJimba

Department of Community and Global Health, Graduate School of Medicine, The University of Tokyo

7-3-1 Hongo, Bunkyo-ku, Tokyo, Japan

Tel: +81-3-5841-3698 / Fax: +81-3-5841-3422 / E-mail: mjimba@m.u-tokyo.ac.jp

Abraham Hodgson

Research and Development Division, Ghana Health Service, Ministry of Health

Accra, Ghana

Tel: 233-3021-684271 / Fax: 233-3021-666808 / E-mail: info@ghsmail.org

Hannah Frimpong

Ghana Health Service Ethical Review Committee, Accra, Ghana

Tel: +233-0302-681109, 233-0302-679323 / Fax: 233-0302-685424 / E-mail: nitadzy@yahoo.com

Regional contacts:

Chairperson of Dodowa HRC IRB

Dodowa Health Research Centre Institutional Review Board, Dodowa, Ghana

Tel: +233-0302-925837 / E-mail: info@dhrc-ghs.org

Chairperson of Kintampo HRC IRB

Kintampo Health Research Centre Institutional Review Board, Kintampo, Ghana

Tel: +233-0352-092037 / E-mail: khrc.iec@kintampo-hrc.org

Chairperson of Navrongo HRC IRB

Navirongo Health Research Centre Institutional Review Board, Navrongo, Ghana

Tel: +233-742-22348 / Fax: 233-742-22348 / E-mail: irb@navrongo.mimcom.net

Informed Consent Form for Women Participants

Study Title:

EMBRACE Implementation Research in Ghana

Principal Investigator:

- MasamineJimba(Department of Community and Global Health, Graduate School of Medicine, The University of Tokyo)
- Abraham Hodgson (Research & Development Division /Ghana Health Service/Ministry of Health)

I, after reading the contents of this study, understand what is expected of me as healthcare provider in the study.

I understand:

1. The purpose and procedure of the study.
2. The consent required of me for completion of the questionnaire.
3. That I will not be placed under any harm or discomfort.
4. That I may refuse to answer any question if I don't want to answer (during or after the study) without any harm or without in any way affecting the health service that I receive.
5. The discussion will be recorded for data analysis, but will be totally deleted after analysis is completed.
6. That any information I provide will be treated in a strictly confidential manner and that I will not be identified in the reporting of the results.

Signature (or thumb print) of the person who gives consent: ________________________
Name of person who gives consent: ________________________________ Date: / /

Signature (or thumb print) of the guardian if the person who receives consent is a minor: __________________
Name of guardian: ____ Date: / /
Witness: I have witnessed the accurate reading of the consent form to the potential participant and the individual has had the opportunity to ask questions. I confirm that the individual has given consent freely.
Name of Witness: ___________________________Signature: ___________________ Date: / /
Person administering consent (field worker): ___________________________ Date: / /

Information Sheet for MNCH Care Providers

Study Title:

EMBRACE Implementation Research in Ghana

Principal Investigator:

- MasamineJimba(Department of Community and Global Health, Graduate School of Medicine, The University of Tokyo)
- Abraham Hodgson (Research & Development Division /Ghana Health Service/Ministry of Health)

Dear Participants,

This study aims to improve maternal, neonatal, and child health status by strengthening the Continuum of Care (CoC) in rural Ghana. In this study, we would like to conduct a questionnaire survey. We won't ask your name in this study. So, your identity will not be disclosed. If you agree to proceed with the study, we will conduct the interview.

This study is approved by the Ethics Committee of the University of Tokyo, the Ghana Health Service (GHS) Ethics Committee, as well as the Ethics Committees of the Kintampo, Dodowa, and Navrongo Health Research Centers. Some of the questions may prove embarrassing for you. However, your participation in this study is entirely voluntary and you may refuse to answer any question if you choose at any time without penalty. The interview will take about **30** minutes. All the information we obtain from you will remain strictly confidential and your responses will never be identified. For the focus group discussions and the in-depth interview, participants' discussions will be recorded for data analysis, but will be totally deleted after analysis is completed.

You may ask any question about the study at this time, and if you have further questions about this study, please do not hesitate to utilize the below contacts.

Masamine Jimba

Department of Community and Global Health, Graduate School of Medicine, The University of Tokyo

7-3-1 Hongo, Bunkyo-ku, Tokyo, Japan

Tel: +81-3-5841-3698 / Fax: +81-3-5841-3422 / E-mail: mjimba@m.u-tokyo.ac.jp

Abraham Hodgson

Research and Development Division, Ghana Health Service, Ministry of Health

Accra, Ghana

Tel: 233-3021-684271 / Fax: 233-3021-666808 / E-mail: info@ghsmail.org

Hannah Frimpong

Ghana Health Service Ethical Review Committee, Accra, Ghana

Tel: +233-0302-681109, 233-0302-679323 / Fax: 233-0302-685424 / E-mail: nitadzy@yahoo.com

Regional contacts:

Chairperson of Dodowa HRC IRB

Dodowa Health Research Centre Institutional Review Board, Dodowa, Ghana

Tel: +233-0302-925837 / E-mail: info@dhrc-ghs.org

Chairperson of Kintampo HRC IRB

Kintampo Health Research Centre Institutional Review Board, Kintampo, Ghana

Tel: +233-0352-092037 / E-mail: khrc.iec@kintampo-hrc.org

Chairperson of Navrongo HRC IRB

Navirongo Health Research Centre Institutional Review Board, Navrongo, Ghana

Tel: +233-742-22348 / Fax: 233-742-22348 / E-mail: irb@navrongo.mimcom.net

Informed Consent Form for MNCH Care Providers

Study Title:

EMBRACE Implementation Research in Ghana

Principal Investigator:

- Masamine Jimba(Department of Community and Global Health, Graduate School of Medicine, The University of Tokyo)
- Abraham Hodgson (Research & Development Division /Ghana Health Service/Ministry of Health)

I, after reading the contents of this study, understand what is expected of me as healthcare provider in the study.

I understand:

7. The purpose and procedure of the study.
8. The consent required of me for completion of the questionnaire.
9. That I will not be placed under any harm or discomfort.
10. That I may refuse to answer any question if I don't want to answer (during or after the study) without any harm or without in any way affecting the health service that I receive.
11. The discussion will be recorded for data analysis, but will be totally deleted after analysis is completed.
12. That any information I provide will be treated in a strictly confidential manner and that I will not be identified in the reporting of the results.

Signature of the person who gives consent: ________________________

Name of person who gives consent: ________________________________ Date: / /

Person administering consent (field worker): ___________________________ Date: / /

Information Sheet for Women Participants on taking photos and movies

Study Title:

EMBRACE Implementation Research in Ghana

Principal Investigator:

- **Masamine Jimba** (Department of Community and Global Health, Graduate School of Medicine, The University of Tokyo)
- **Abraham Hodgson** (Research & Development Division /Ghana Health Service/Ministry of Health)

Dear Participants,

This study aims to improve maternal, neonatal, and child health status by strengthening the Continuum of Care (CoC) in rural Ghana. In this study, we would like to take photos and movies of you and your baby during the interview. The photos and the movies may be shown at public place, such as national and international conferences. We won't present your personal information in the photos and the movies. If you agree, we will conduct the interview with taking photos and movies.

This study is approved by the Ethics Committee of the University of Tokyo, the Ghana Health Service (GHS) Ethics Committee, as well as the Ethics Committees of the Kintampo, Dodowa, and Navrongo Health Research Centers. Some of the questions may prove embarrassing for you. However, your participation in this study is entirely voluntary and you may refuse to answer any question and to be taken photos and movies if you choose at any time without penalty. The interview will take about 30 minutes. The information we obtain from you may be exposed at public place, but your responses will never be identified. The phots and movies will be totally deleted after the purpose for using them is achieved.

You may ask any question about the study at this time, and if you have further questions about this study, please do not hesitate to utilize the below contacts.

Masamine Jimba

Department of Community and Global Health, Graduate School of Medicine, The University of Tokyo

7-3-1 Hongo, Bunkyo-ku, Tokyo, Japan

Tel: +81-3-5841-3698 / Fax: +81-3-5841-3422 / E-mail: mjimba@m.u-tokyo.ac.jp

Abraham Hodgson

Research and Development Division, Ghana Health Service, Ministry of Health

Accra, Ghana

Tel: 233-3021-684271 / Fax: 233-3021-666808 / E-mail: info@ghsmail.org

Hannah Frimpong

Ghana Health Service Ethical Review Committee, Accra, Ghana

Tel: +233-0302-681109, 233-0302-679323 / Fax: 233-0302-685424 / E-mail: nitadzy@yahoo.com

Regional contacts:

Chairperson of Dodowa HRC IRB

Dodowa Health Research Centre Institutional Review Board, Dodowa, Ghana

Tel: +233-0302-925837 / E-mail: info@dhrc-ghs.org

Chairperson of Kintampo HRC IRB

Kintampo Health Research Centre Institutional Review Board, Kintampo, Ghana

Tel: +233-0352-092037 / E-mail: khrc.iec@kintampo-hrc.org

Chairperson of Navrongo HRC IRB

Navirongo Health Research Centre Institutional Review Board, Navrongo, Ghana

Tel: +233-742-22348 / Fax: 233-742-22348 / E-mail: irb@navrongo.mimcom.net

Informed Consent Form for Women Participants on taking photos and movies

Study Title:

EMBRACE Implementation Research in Ghana

Principal Investigator:

- **Masamine Jimba (Department of Community and Global Health, Graduate School of Medicine, The University of Tokyo)**
- **Abraham Hodgson (Research & Development Division /Ghana Health Service/Ministry of Health)**

I, after reading the contents of this interview, understand what is expected of me.

I understand:

13. **The purpose of interview and taking photos and movies of me and my baby.**
14. **The consent required of me for taking photos and movies of me and my baby during interviews.**
15. **That I will not be placed under any harm or discomfort.**
16. **That I may refuse to be taken photos and movie without any harm or without in any way affecting the health service that I receive.**
17. **The photos and interviews will be shown at public place, but will be totally deleted after the purpose of using them is achieved.**
18. **That any information I provide may be exposed in public place, but that I will not be identified in the presentation for the photos and the movies.**

Signature (or thumb print) of the person who gives consent: ________________________

Name of person who gives consent: ________________________________ Date: / /

Signature (or thumb print) of the guardian if the person who receives consent is a minor: __________________

Name of guardian: ____ Date: / /

Witness: I have witnessed the accurate reading of the consent form to the potential participant and the individual has had the opportunity to ask questions. I confirm that the individual has given consent freely.

Name of Witness: ___________________________Signature: ___________________ Date: / /

Person administering consent (field worker): ___________________________ Date: / /

Information Sheet for MNCH Care Providers on taking photos and movies

Study Title:

EMBRACE Implementation Research in Ghana

Principal Investigator:

- **Masamine Jimba**
  (Department of Community and Global Health, Graduate School of Medicine, The University of Tokyo)
- **Abraham Hodgson (Research & Development Division /Ghana Health Service/Ministry of Health)**

Dear Participants,

This study aims to improve maternal, neonatal, and child health status by strengthening the Continuum of Care (CoC) in rural Ghana. In this study, we would like to take photos and movies of you during the interview. The photos and the movies may be shown at public place, such as national and international conferences. We won't present your personal information in the photos and the movies. If you agree, we will conduct the interview with taking photos and movies.

This study is approved by the Ethics Committee of the University of Tokyo, the Ghana Health Service (GHS) Ethics Committee, as well as the Ethics Committees of the Kintampo, Dodowa, and Navrongo Health Research Centers. Some of the questions may prove embarrassing for you. However, your participation in this study is entirely voluntary and you may refuse to answer any question and to be taken photos and movies if you choose at any time without penalty. The interview will take about 30 minutes. The information we obtain from you may be exposed at public place, but your responses will never be identified. The phots and movies will be totally deleted after the purpose of using them is achieved.

You may ask any question about the study at this time, and if you have further questions about this study, please do not hesitate to utilize the below contacts.

Masamine Jimba

Department of Community and Global Health, Graduate School of Medicine, The University of Tokyo

7-3-1 Hongo, Bunkyo-ku, Tokyo, Japan

Tel: +81-3-5841-3698 / Fax: +81-3-5841-3422 / E-mail: mjimba@m.u-tokyo.ac.jp

Abraham Hodgson

Research and Development Division, Ghana Health Service, Ministry of Health

Accra, Ghana

Tel: 233-3021-684271 / Fax: 233-3021-666808 / E-mail: info@ghsmail.org

Hannah Frimpong

Ghana Health Service Ethical Review Committee, Accra, Ghana

Tel: +233-0302-681109, 233-0302-679323 / Fax: 233-0302-685424 / E-mail: nitadzy@yahoo.com

Regional contacts:

Chairperson of Dodowa HRC IRB

Dodowa Health Research Centre Institutional Review Board, Dodowa, Ghana

Tel: +233-0302-925837 / E-mail: info@dhrc-ghs.org

Chairperson of Kintampo HRC IRB

Kintampo Health Research Centre Institutional Review Board, Kintampo, Ghana

Tel: +233-0352-092037 / E-mail: khrc.iec@kintampo-hrc.org

Chairperson of Navrongo HRC IRB

Navirongo Health Research Centre Institutional Review Board, Navrongo, Ghana

Tel: +233-742-22348 / Fax: 233-742-22348 / E-mail: irb@navrongo.mimcom.net

Informed Consent Form for MNCH Care Providers on taking photos and movies

Study Title:

EMBRACE Implementation Research in Ghana

Principal Investigator:

- **Masamine Jimba(Department of Community and Global Health, Graduate School of Medicine, The University of Tokyo)**
- **Abraham Hodgson (Research & Development Division /Ghana Health Service/Ministry of Health)**

I, after reading the contents of this study, understand what is expected of me as healthcare provider in the study.

I understand:

1. **The purpose of interview and taking photos and movies of me.**
2. **The consent required of me for taking photos and movies of me and during interviews.**
3. **That I will not be placed under any harm or discomfort.**
4. **That I may refuse to be taken photos and movie without any harm or without in any way affecting the health service that I receive.**
5. **The photos and interviews will be shown at public place, but will be totally deleted after the purpose for using them is achieved.**
6. **That any information I provide may be exposed in public place, but that I will not be identified in the presentation of the photos and the movies.**

Signature of the person who gives consent: ________________________

Name of person who gives consent: ________________________________ Date: / /

Person administering consent (field worker): ___________________________ Date: / /

In-depth Interview Guide for Women

Overview

This is a guide to conduct an in-depth interview (IDIs) with women who participated in the EMBRACE intervention. The participants will be selected by convenience sampling. They will be recruited from Dodowa, Kintampo, and Navrongo intervention areas to respond to issues such as their attitude to continuum of care, health seeking behaviors, and health status. The interview will last for about 30 minutes, and the responses will be recorded using recorders to completely transcribe the voice data. After transcription, the voice data will be deleted.

Objectives

To supplement the information collected from the quantitative survey

To grasp what made mothers to follow/unfollow the continuum of care

Procedure

Welcome, background information and introduction

Obtain informed consent

Fill out background information sheet

Conduct IDI

Ghana EMBRACE Implementation Research – Intervention Evaluation

In-depth Interview Guide for Women

Information on data collection team

Interviewer's name:	

Note-taker's name:	

IDI venue: District	
Community	

IDI date (DD/MM/YY)	

IDI start time	AM / PM :

IDI end time	AM / PM :

Ghana EMBRACE Implementation Research – Intervention Evaluation

Background Information Sheet for IDI Participants

Interview Number:

IDI-	IDINB

Date of interview (DD/MM/YY):

						DAT

1.1 Name of the village/community:

	VIL

1.2 Location of residence:

0. Residence	1.Farm hut	RES

1.3 Age of the mother: (“88” when the respondent do not know her age)

	AGE

1.4 Age of the infant (months): (“88” when the respondent do not know the age of her infant)

months	IAGE

1.5 Gender of the infant:

0. Male	1.Female	ISEX

1.6 Marital status:

1. Married	2. Unmarried	MAR

1.7 Education level:

1.No education	2. Some years in **primary** school	3. Graduate **primary**	EDU
4. Some years in secondary school	5. More than secondary level		

1.8 Number of family members living together (a number except the respondent):

	NFAM

1.9 Number of children living together:

	NCHI

1.10 Family members living together (multiple choice):

1. Husband	2. Farther-in-law	3. Mother-in-law		FMEM
4. Father	5. Mother	6. Children	7. Others	

1.11 Occupation of respondent

1. Farmer/laborer/domestic worker	2. House wife	ROC
3. Profession: teacher, nurse,	4. Employed tradesman, driver	

accounts, administrator, etc	without own car, builder, etc.
5. Trader/ businessman / driver with own car etc.	6. Clerical / secretarial
7. Other	

Ghana EMBRACE Implementation Research – Intervention Evaluation

Section 1: Socio-demographic characteristic and interview information

Self-introduction

Section 2: Continuum of Care (CoC)

Facilitator "I would like to ask about the CoC."

Do you know CoC? **Probe:** What are the contents of CoC? Why it is important?

(For those who completed CoC) What was your motivation for going antenatal care 4 times, giving birth at health facility, and going to PNC after birth? **Probe**: What is the benefit of having CoC?

(For those who did not accomplish any of CoC) What made you not receive antenatal care, delivery at facility, and postnatal care?

Section 3: CoC card

Facilitator "I would like to ask about the CoC card."

What do you think of CoC card? **Probe:** which parts do you like?

Did you talk about CoC card with others? **Probe:** did you talk with other pregnant women or husband? What did you talk?

Do you find any changes in this pregnancy, compared with previous one(s)? Probe: what? How? Why?

What does it mean to collect all the stars?

There were two different colours of stars in use (Gold and Orange). What is the difference between the gold stars and the orange stars?

Section 4: Delivery and postnatal care

Facilitator "I would like to ask you about delivery and postnatal care"

Please tell me your experience from the start of labour to giving birth.

What do you think about giving birth at home? What do you think about giving birth at health facility? **Probe:** How important is a health worker's assistance (doctor, nurse, and midwife) during delivery?

What do you think about staying at health facility after birth?

Probe: If you stayed at health facility after deliver, what was the benefit? What was the difficulties?

What do you think of the postnatal care service?

Probe: If you stayed at health facility after deliver, what were the benefits?

What were the difficulties?

Have you received postnatal care at home? If yes, what do you think of that?

What do you think of your child's health status?

Ghana EMBRACE Implementation Research – Intervention Evaluation

In-depth interview guide for health workers

Overview:

This is a guide to conduct in-depth interview (IDI) with trained health workers: doctors; nurses; midwives; and district public health nurse from community to district hospital level. Participants will be recruited from the Dodowa, Kintampo, and Navrongo Research Center Demographic surveillance areas to obtain information on issues related to changes in their attitudes and mothers' attitudes to continuum of care, as well as changes in health status of mothers and children. The interview will last for about 45 minutes, and their responses will be recorded using recorders to completely transcribe the voice data. After transcription, the voice data will be deleted.

Objectives:

To supplement the information collected from the quantitative survey

To obtain insight from health worker's side into the changes in attitudes to the continuum of care

To obtain insight from health worker's side into the changes in health status of mothers and children

Procedure

Obtain informed consent

Fill out background information sheet

Conduct IDI

Section 1: Socio-demographic characteristics and interview information

Fill out background information sheet

In-depth Interview Number:

	IDINBHW

Date of interview (DD/MM/YY):

						DAT

Starting time (HH/MM):

				STATM

Termination time (HH/MM):

				TRMTM

1.1 Type of participant

1.District level health officer	2. Obstetricians in hospital	3. Midwives in health centre	TYPE
4. CHOs and CHN	5. TBA		

1.2 Name of village

	VIL

1.3 Age: ("88" when the respondent do not know her age)

	AGE

1.4 Gender:

0. Male	1.Female	SEX

1.5 Education level:

1.No education	2. Some years in **primary** school	3. Graduate **primary**	EDU
4. Some years in secondary school	5. More than secondary level		

1.6 Years working at this position???for the actual post:

	YRSWP

Interview guide

Section 2: Continuum of care

What changes have you found in mothers response to services since starting intervention (since introducing CoC card)?

Probe for:

What changes have you found in your service delivery as health workers since starting intervention (since introducing CoC card)?

How is interaction (relationship) between health workers and mothers since starting intervention (CoC card)?

What do you think of continue using CoC card?

What are mothers' reaction towards staying on at the health facility for 24 hours after delivery?

What is your opinion about the achievement of continuum of care from pregnancy to the postnatal period in this facility?

Probe for: Achievement of continuum of care from pregnancy to the postnatal period and reasons for lapses?

Probe: for positive and negative responses and for the period from pregnancy to the postnatal period when the continuum of care disrupted?

How would you assess the quality of maternal and child health services in your facility?

How acceptable are maternal and infant health services in your facility?

Probe: for positive and negative res
