# Supplementary material for: Evaluation of a package of continuum of care interventions for improved maternal, newborn, and child health outcomes and service coverage in Ghana: A cluster-randomized trial
Source: PLoS Med. 2021 Jun 25;18(6):e1003663. doi: 10.1371/journal.pmed.1003663 (PMC8232410; doi:10.1371/journal.pmed.1003663)
Supplement: Attachment — Submitted filename: Response_to_reviewer_v06.docx [file pmed.1003663.s005.docx]

Thank you very much for taking the time to review our manuscript and allow us to make revisions. A point-by-point response to your comments is listed below.

Instructions

| 1. In revising the manuscript for further consideration, your revisions should address the specific points made by each reviewer and the editors. Please also check the guidelines for revised papers at http://journals.plos.org/plosmedicine/s/revising-your-manuscript for any that apply to your paper. In your rebuttal letter you should indicate your response to the reviewers' and editors' comments, the changes you have made in the manuscript, and include either an excerpt of the revised text or the location (eg: page and line number) where each change can be found. Please submit a clean version of the paper as the main article file; a version with changes marked should be uploaded as a marked up manuscript. | We revised the manuscript according to the suggestions and comments from the editor and reviewers. Particularly, we added an Author Summary (page iv) and listed the abbreviations before the main text. |
| --- | --- |
| 2. In addition, we request that you upload any figures associated with your paper as individual TIF or EPS files with 300dpi resolution at resubmission; please read our figure guidelines for more information on our requirements: http://journals.plos.org/plosmedicine/s/figures. While revising your submission, please upload your figure files to the PACE digital diagnostic tool, https://pacev2.apexcovantage.com/. PACE helps ensure that figures meet PLOS requirements. To use PACE, you must first register as a user. Then, login and navigate to the UPLOAD tab, where you will find detailed instructions on how to use the tool. | We uploaded Figures 1 and 2 in TIF format with 300dpi resolution, converted using the PACE digital diagnostic tool. |

Requests from the editors

| 1. To your data statement, for readers interested in inquiring about access to study data please add contact details for a non-author contact. | We added a non-author institutional contact as follows:  “For contact with the Principal Investigator, send an email to the following institutional email address of Department of Community and Global Health, Graduate School of Medicine, the University of Tokyo: [ich@m.u-tokyo.ac.jp](mailto:ich@m.u-tokyo.ac.jp).” (Line 526-) |
| --- | --- |
| 2. In the abstract and throughout the paper, please add p values alongside 95% CI, where available. | We added p-values for the results of the mixed-effects regression analysis in the abstract and the main text. |
| 3. Please add summary details to your abstract of the number of sites, women and women-child pairs studied, and if possible aggregate demographic details for the participants. | We modified the abstract, while staying within the word count limit, as follows:  Clarify the number of sites, we modified the first sentence of the Methods and Findings section as follows:  “This cluster randomized controlled trial (ISRCTN: 90618993) was conducted at three Health and Demographic Surveillance Systems Sites in Ghana.” (Line 6-)  For the women and women-child pairs studies, in addition to disaggregated numbers by the arm and phase, we added the descriptions in the Results section:  “For health-service and morbidity outcomes, 2,970 woman-child pairs were assessed: 1,480 from the baseline survey and 1,490 from the follow-up survey. Additionally, 33,322 cases were assessed for perinatal and neonatal mortality outcomes 39,205 cases for maternal mortality outcome.” (Line 22-) |
| 4. Where you state the study dates in the abstract, we suggest adding additional details of the before and subsequent survey periods, along with the inclusion criteria. | We added the inclusion criteria along with the survey periods as follows:  “Eligibility criteria were as follows: women who gave birth or had a stillbirth from September 1, 2012, to September 30, 2014 (before the trial period), from October 1, 2014, to December 31, 2015 (during the trial period), or from January 1 to December 31, 2016 (after the trial period).” (Line 15-) |
| 5. Please combine the "methods" and "findings" subsections of your abstract. The final sentence of the new combined subsection should quote 2-3 of the study's main limitations. | In the abstract, the methods and results were combined to create the new section, “Methods and Findings.” The section includes the following regarding the study’s limitations:  “This study was conducted under real-world settings. Possible limitations included differences in the type and scale of health facilities and the size of sub-districts, contamination for intervention effectiveness due to the geographic proximity of the arms, and insufficient number of cases for the mortality assessment.” (Line 35-) |
| 6. Please state the study's primary and other outcomes early in the "methods and findings" subsection of your abstract. | The revised Methods and Findings section includes the following statement:  “The primary outcome was CoC completion by a woman-child pair, defined as receiving antenatal care four times or more, delivery assistance from a skilled birth attendant, and postnatal care three times or more. Other outcomes were the morbidity and mortality of women and children.” (Line 7-) |
| 7. Please begin the "Conclusions" subsection of your abstract with "In this study, we found that ..." or similar. | In the abstract, the Conclusions section was modified as follows:  “This study found that an integrated package of CoC interventions increased CoC completion, decreased maternal complications requiring hospitalization during pregnancy, and maternal mortality after the trial period. It did not find evidence regarding the reduction of perinatal and neonatal mortality.” (Line 40-) |
| 8. After the abstract, please add a new and accessible "author summary" section in non-identical prose. You may find it helpful to consult one or two recent research papers published in PLOS Medicine to get a sense of the preferred style. | We added an Author Summary section, including “Why Was This Study Done?” “What Did the Researchers Do and Find?” and “What Do These Findings Mean?” (Page iv) |
| 9. Please remove the "Role of the funding source" section from the end of the methods section. In the event of publication, information on funding will appear in the article metadata, via entries in the submission form. Similarly, please remove information on competing interests from the end of the main text. | As you have requested, we removed the “Roles of funding source” subsection from the Methods section. |
| 10. Throughout the text, please ensure that reference call-outs fall consistently before punctuation, and remove spaces from within the square brackets (e.g., "... (LMICs) [1,2]."). | We checked all the reference call-outs in the manuscript to ensure that they are placed before the punctuation and removed any spaces within the square brackets. |
| 11. Thank you for including a completed CONSORT checklist. Please adapt the checklist so that individual items are referred to by section (e.g., "Methods") and paragraph number rather than by line or page numbers, as the latter generally change in the event of publication. | We modified the CONSORT checklist to include section and paragraph numbers. |
| 12. Please remove the published study protocol from the supplementary files - a reference will suffice. | We removed the published study protocol from the supplementary files. |

Reviewer #1

| 1. Shibanuma et al present a report on a large cluster randomised trial of a package of health service interventions to improve the continuity of care for pregnant women and their babies in Ghana. This review considers the use of statistics in the paper.   Overall, the study design, statistical analyses, their presentation and interpretation, are good. I do have a number of observations, however. Whist they are many, each of these is relatively minor, and if dealt with, should improve the paper. | Thank you very much for your valuable advice. We made corrections and modifications to our statistical analysis accordingly. |
| --- | --- |
| 2. According to the protocol paper, the primary outcome is the CoC completion rate, but this paper does not specify which outcome is primary. Since this was prespecified, it should really be presented first. The abstract, and the results section, both present mortality first. | We modified the entire manuscript to mention CoC completion as the primary outcome and present it before other outcomes throughout the manuscript. |
| 3. Also in the abstract, it is noted that perinatal and neonatal mortality were not significantly reduced in the intervention arm, but AORs and CIs are not provided - should they be? | We did not include AORs and CIs for perinatal and neonatal mortality due to the limited word count for the abstract.  The relevant results are presented in the main text as follows:  “Perinatal mortality was 130 cases among 5,961 live births in the intervention arm and 104 among 4,138 in the control arm during the trial period (p=0.303; In the DiD analysis, it decreased by 19% or 5 points) and 71 among 3,354 in the intervention arm and 64 among 2,824 in the control arm after the trial period (p=0.745; In the DiD analysis, it decreased by 13% or 4 points). Neonatal mortality was 73 cases among 5,961 live births in the intervention arm and 57 among 4,138 in the control arm during the trial period (p=0.510; In the DiD analysis, it decreased by 3% or 0 points) and 30 among 3,354 in the intervention arm and 25 among 2,824 in the control arm after the trial period (p=0.970; In the DiD analysis, it increased by 10% or 1 point).”  (Line 344-) |
| 4. The fact that sub-districts that contained a district hospital were automatically allocated to the intervention group raised some concerns when I first read it, but after reading the discussion, I can see that this was perhaps a necessary decision. I think the rationale for doing this should be stated in the methods, and not left until the discussion. Also, I am guessing that this relates to 3 intervention and 3 control sub-districts (since they say there was one district hospital per district), but it would help if the authors were absolutely clear on how many sub-districts were affected. | The rationale provided for allocating a sub-district where a district hospital was located to the intervention arm was modified, in addition to the existing statement in the second paragraph of the subsection “Randomization and masking” in the Methods section, as follows:  “A sub-district was immediately allocated as an intervention arm if it had the only district hospital in a district.  This method to allocate the intervention arm was used because women had autonomy in health facility choice in the study area and many women were expected to choose the district hospital, regardless of their arms, especially for delivery. The district hospital also served as the referral hospital for the whole district. In addition, it played a leadership role in managing MNCH services at the district level.” (Line 179-) |
| 5. The sample size sections could be better. Firstly, they are in the wrong order, if CoC completion was the primary outcome. Secondly, they do not give the assumed average cluster size, or whether the calculation assumes equal cluster sizes or allows for variable cluster sizes. Thirdly, the protocol paper states that the required sample size for the mortality analysis was 15,000, not 30,000 as stated in this paper. | In the subsection of “Sampling of women and children,” the explanation of the sample size calculation for health-service and morbidity outcomes (including CoC completion) was moved to the first paragraph, followed by the explanation of the sample size calculation for mortality outcomes. We also that the ICC of health-service outcomes was assumed to be 0.02675 (average cluster size was 102), and that the ICC of mortality outcomes was 0.0007256 (average cluster size was 102). The required sample size stated in the protocol paper should have been 15,000 for the baseline and follow-up survey, respectively (in the third paragraph, “Sample size” in Page 8 of the protocol paper). |
| 6. In the statistical methods, chi-square tests are reportedly used to compare outcomes, but this is not suitable for a cluster randomised trial. Reporting analyses without adjustment for covariates is fine, but any analysis of a cluster randomised trial should account for the clustering. The mixed effects logistic regression analysis is more appropriate. However, the protocol paper states that a GEE method was to be used, so the authors should justify their decision to use an alternative method. | We reanalyzed the outcomes in Tables 2 and 3 using Rao-Scott cluster-adjusted chi-squared tests, via Stata 15 MP’s svy: tabulate command. In addition, we explained our reason for choosing a mixed-effects model, instead of using GEE, as we acknowledged the importance of accounting for random effects at the two levels (sub-districts and clusters), as follows:  “Mixed-effects models were used, instead of generalized estimating equations as stated in the protocol paper, as the model accounting for random effects at the two levels (HDSS site and community).” (Line 266-). |
| 7. Also reported in the protocol paper is that the analyses will be by intention to treat, and I think that is worth adding to this paper. The protocol paper states that all randomised participants will be included in analyses, regardless of missing data. Whilst in my opinion, this is not a requirement of ITT, it is nevertheless a worthwhile thing to do. However, it should be made clear that this has been done, and what assumptions or specific methods were used to deal with missing outcome data. If, on the other hand, this was not done, then the authors should again justify this deviation from their original plan. | In the first paragraph of the subsection of “Statistical analysis,” we added the following explanation:  “In this study, intervention effectiveness was estimated using the intention-to-treat approach.” (Line 261-)  We did not perform analysis based on the existence of missing data for the following reasons:  “An analysis of missing data was not performed for health-service and morbidity outcomes because the woman-child pairs surveyed did not have missing information for the outcomes or covariates. Additionally, this analysis was not performed for mortality assessment as the assessment did not have information on missing observations and covariates were not available.” (Line 277-) |
| 8. The mortality outcomes were collected in three phases (before, during and after the trial), but no mention is made of the analysis of these outcomes during the trial, other than in the tables; in the main text of the paper, only the post-trial period mortality results are reported. Also, in the footnote to Table 4, it seems as though each post-randomisation time point was analysed separately. Would it be better to analyse both time points within the same regression model, i.e. pooling data from three time points, modelling the time effect as a 3-level categorical variable, and estimating the intervention effects at each time point through the addition of time-by-intervention interactions? | In the main text, we added explanations regarding the results for the mortality outcomes, including that they were not significant. The multilevel mixed-effects regressions were conducted by pooling the three time points (having three time point variables, the variable for arms, and the two post-intervention-time-by-intervention interactions). We added further clarification as follows:  “Regarding mortality outcomes, pooled data were used before, during, and after the trial periods without controlling for confounders because of unavailability in HDSS data. Intervention effectiveness was estimated using the two interaction terms (arm and during the trial period; arm and after the trial period).” (Line 274-) |
| 9. The analyses presented in Supplementary Table 1 seem odd. I do not see the value of comparing those who live in or close to an intervention area, with those living in a control area but not close to an intervention area; it would make more sense to consider the 3-level categorical variable of living in an intervention area vs. living close to an intervention area vs. not living close to an intervention area. When looking at the type of the nearest facility, all facilities in control areas are treated the same. The pattern of differences between types of facility seen in intervention areas may be seen in control areas as well, but it is not possible to tell from the analysis that is presented. The third analysis breaks down the intervention sub-districts according to the type of interventions available; it is not possible to split control group sub-districts in the same way, so this analysis makes more sense. | We modified the first analysis of Supplementary Table 1 by setting three categories (the three-level categorical variable of living in an intervention area vs. living close to an intervention area vs. not living close to an intervention area), as suggested by the reviewer.  Regarding the second analysis, we modified the analysis regarding type of the nearest health facility by setting up a categorical variable for facility type for both intervention and control arms.  Regarding the third analysis, we maintain the present analysis, as suggested by the reviewer. |
| 10. The remainder of Supplementary Table 1 is different, in that it is looking at intervention effects within subgroups of sub-districts. However, these analyses would be improved if they used interaction terms to test for differences in intervention effects between subgroups. It is not sufficient to estimate intervention effects within subgroups and draw conclusions form which are statistically significant. Statistical evidence of different intervention effects between subgroups is required to draw any conclusions. | We modified the models in the latter half of Supplementary Table 1 (*Sub-district characteristics*) by pooling all the observations and constructing a categorical variable that combines sub-district characteristic and arm. Then, the models included an interaction term between the timing (before or during the trial period) and the categorical variable. |
| 11. In Figure 2, for the mortality outcomes, does it make sense to state the number of women or mother-child pairs who were "surveyed"? No one was surveyed, if the data were taken from administrative records. Also, if these data were only available in two of the three HDSS sites involved in the study, is it fair to say that 32 sub-districts were included in these analyses? | We modified Figure 2 to clarify that women or mother−child pairs were assessed (not surveyed) from a total of 24 sub-districts (not 32 sub-districts) for the mortality outcomes. |
| 12. The footnote of Table 2 states that chi-square tests were used to compare means between arms. This is not correct - a chi-square test compares proportions or percentages between groups, though as stated above, simple chi-square tests are not appropriate for a cluster randomised trial. | We corrected the descriptions in the footnotes of Tables 2 and 3 as follows:  Rao-Scott Cluster adjusted chi-squared test was used to compare the proportions of an outcome by arm. |
| 13. The footnote to Table 4 reports that the models were adjusted for several covariates. It is slightly surprising that so much information is available from administrative records, and that it is complete. I would assume a fairly high level of missingness in administrative data - if that is the case, how much missing data was there, and how was it dealt with? | We modified the footnote to Table 5 (formally Table 4) to report that models for mortality outcomes were not controlled for covariates, as follows:  Mortality outcomes were analyzed by pooling three datasets before the study periods, during the study periods, and after the study periods and using the model without other covariates due to unavailability of these covariates. |
| 14. In the CONSORT checklist, I note that the ICCs for the data presented in the paper are not provided. Also, the checklist suggests reporting both relative and absolute effect sizes; was this done? As far as I can tell, the results are exclusively reported as relative effects. | In Tables 2 and 3, we reported the rate ratio and absolute risk difference in addition to the raw number in the outcome assessment. We added ICCs for all the models in Table 5. Also, we added NNTs for models with significant DiD estimators. |

Reviewer #2

| 1. Thank you for this manuscript, which describes an important study to look at the effectiveness of intervention to support completion of the continuum of care for mothers and children in a selection of health facilities in Ghana. As a reviewer, I am not qualified to scrutinise the statistics and methodological approaches applied and would ask that an appropriate reviewer with strong epi or med-stats background offers their comment on this article. | Thank you very much for your kind comments. |
| --- | --- |
| 2. Some minor changes to English language would improve readability. Some specific areas of note as follows:   Background section: p1 line 24: suggest rephrase - sentence doesn't read very easily p1 link 26-28: suggest use the word "however" instead of while p2 line 45-48 : Sentence is too long - suggest to break up. | We have modified the relevant sentences as follows:  However, no study has evaluated the effectiveness of interventions that were intended to improve CoC under the real-world setting.  “Ghana has demonstrated an extensive improvement in MNCH health indicators, although the current trend of improvement might not ensure the global targets set forth in the SDGs are achieved, particularly for maternal health.” (Line 114-)  The following sentences were reorganized based on the two reviewers’ comments:  “The retention is part of Ghana’s MNCH guidelines. The coverage of PNC can also be improved by providing PNC at women’s homes, although an adequate number of trained community health workers would be necessary to conduct timely home visits [25-27]. In Ghana, community health workers are designated to conduct home visits [28]. Nonetheless, prior to this study, retention and home visits had yet to be largely adhered to in the study areas, mainly owing to the lack of physical infrastructure and human resources.” (Line 127-) |
| 3. Methods: p4 first few paragraphs on district hospitals - the description of this is a bit convoluted and does bring into question the randomisation process overall. I think this is then well-described in the discussion, but still a bit confusing here. | We modified the justification we provided for the allocation of a sub-district where a district hospital was located to the intervention arm, as follows:  “This method to allocate the intervention arm was used because women had autonomy in health facility choice in the study area and many women were expected to choose the district hospital, regardless of their arms, especially for delivery. The district hospital also served as the referral hospital for the whole district. In addition, it played a leadership role in managing MNCH services at the district level.” (Line 181-) |
| 4. Results section: The first paragraph of the results is difficult to read and unclear. p7 line 221-225 Some confusing expressions here - I think less in the formal marriage refers to someone who is either unmarried or single - correct? In the health insurance - more likely to have health insurance? | The first paragraph in the Results section was extensively rewritten. (Line 332-)  Regarding “less in the formal marriage” we added an explanation as follows:  …less in the formal marriage (more cohabitating, divorced, separated, widowed, or never married)… (Line 356-)  Regarding the health insurance, we modified the explanation as “have joined join the health insurance scheme.” (Line 357) |
| 5. In the discussion would be helpful to have some discussion about how this project links to other efforts to scale up emergency obstetric care and postnatal care of newborns. | This project was conducted independently with other initiatives for the improvement of emergency obstetric care and overall quality improvement of MNCH services. To clarify this, we added the following explanation to the Discussion section:  “The scope of this intervention did not include improvements in emergency obstetric care or the quality of MNCH services during ANC, delivery, and PNC. Thus, enhancing access to MNCH services should be made in line with an improvement in emergency obstetric care and the quality of MNCH services.”  (Line 467-) |

Reviewer #3

| 1. Thanks for submitting a very important trial of a bundle of interventions to reduce maternal and perinatal morbidity and mortality. I have a few comments that may be useful in improving the manuscript. | Thank you very much for your valuable comments. |
| --- | --- |
| 2. Abstract Control group is not defined Conclusions could be boldly linked to results | We added more explanation of the control arm as follows:  “In the control arm, women received routine services only.” (Line 14-)  We also modified the concluding statement in the abstract and the first sentence in the Conclusion section of the main text, as follows:  “This study found that an integrated package of CoC interventions increased CoC completion, decreased maternal complications requiring hospitalization during pregnancy, and maternal mortality after the trial period. It did not find evidence regarding the reduction of perinatal and neonatal mortality.” (Line 40-) |
| 3. Introduction Page 1: Line 15: Consider 'give birth' rather than 'deliver newborns' | We modified this in the main text as follows:  …give birth with assistance by a skilled birth attendant (SBA)… (Line 101-) |
| 4. How was the pre-pregnancy component of COC measured in your study? Page 1: Line 24-25: This clain is not accurate. See systematic review published in 2016: Kikuchi K, Okawa S, Zamawe COF, Shibanuma A, Nanishi K, Iwamoto A, et al. (2016) Effectiveness of Continuum of Care—Linking Pre-Pregnancy Care and Pregnancy Care to Improve Neonatal and Perinatal Mortality: A Systematic Review and Meta-Analysis. PLoS ONE 11(10): e0164965. <https://doi.org/10.1371/journal.pone.0164965> | Thank you very much for referring to our previous works. As we noted in the second paragraph of the Background section, we recognize that CoC starts in the pre-pregnancy stage. However, we focused on the pregnancy, delivery, and post-partum stages in this study. To clarify that we modified the justification of this study, we added the following to the main text:  “Health promotion starting from the pre-pregnancy stage could improve perinatal and neonatal mortality [6]. However, no study to date has evaluated the effectiveness of interventions develop to ensure access to ANC, SBA delivery, or PNC under real-world settings.” (Line 110-) |
| 5. Page 1 line 29: Global MMR target is 70/100, 000 but no country should be more than 140/100, 000., so this statement as it ideas not give a true picture of how much behind Ghana is. Either compare with the country level target of 140, 000/100, 000 or compare the Ghanaian figure with Africa regional average. | We modified the presentation of mortality data by comparing them with country-level targets, as follows:  “In Ghana, at the time this study began, maternal mortality ratio was 319 out of 100,000 live births in 2015 although the country’s target in 2015 was 190. Likewise, under-five mortality rate was 59 out of 1,000 live births in 2016 although the country’s target in 2015 was 42.” (Line 116-) |
| 6. Page 1, line 31: Coverage of PNC is unclear, is this 2 visits, 1 visit? If the coverage was based on a visit within 24 hours, say so. | We modified how this is explained in the main text to clarify that the indicator measured PNC three times, as follows:  “CoC completion rate among woman-child pairs, or the coverage of receiving all of ANC four times, SBA delivery, and PNC three times, was 8%, while the coverage of ANC alone was 86% and that of SBA delivery alone was 76% in 2014.” (Line 119-) |
| 7. Page 2 line 35, please rephrase to improve clarity. Line 36 these coverage refers to one visit within 24 hours? Is this feasible? Do Ghana CMWs visit homes within 24hours of birth? Please rephrase line 35-38, so that your message is factually correct. | We modified our explanation of the importance of community health workers providing home-visit PNC, as follows:  “PNC can be provided either at a health facility or by community outreach in women’s homes [24-26]. Women and newborns can receive PNC at a health facility if they are retained there longer after delivery [24]. The retention is part of Ghana’s MNCH guidelines. The coverage of PNC can also be improved by providing PNC at women’s homes, although an adequate number of trained community health workers would be necessary to conduct timely home visits [25-27]. In Ghana, community health workers are designated to conduct home visits [28]. Nonetheless, prior to this study, retention and home visits had yet to be largely adhered to in the study areas, mainly owing to the lack of physical infrastructure and human resources.” (Line 124-) |
| 8. Page 2 line 41 to 49 should be in the methods, not introduction. The interventions should be better described in the methods. | We removed the descriptions of the interventions from the Background section, as they are explained in the Methods. |
| 9. The CoC card is essentially a one page record of CoC intervention received including health education? If this is correct, please rephrase to accurately describe what the CoC card is and what it was used for. | We removed the description on the CoC Card in the Background section and retained the explanation in the Methods section, as follows:  “For A-1, women who received care in a health facility received the one-page CoC card, used to schedule subsequent visits to receive ANC, SBA delivery, and PNC. CoC Card was also used to record the visiting history, the receipt of key care components during ANC and PNC, and complications.” (Line 197-) |
| 10. Page 3, line 68-74 typically this comes last in the methods, just before the results section. | We moved the description of ethical considerations to the end of the Methods section. |
| 11. Page 4: The 32 sub-districts were in how many districts? The randomisation process, intervention and control clusters is very unclear. It will be difficult to replicate this study with the information provided. | We clarified the number of sub-districts as follows:  “These sites had six districts and 36 sub-districts in total.” (Line 146)  “Among the 36 sub-districts at the three collaborating HDSS sites, 32 were included.” (Line 173-)  We modified all descriptions of randomization and masking based on the reviewers’ comments, particularly on matching the sub-districts and the treatment of sub-districts where a district hospital was located.  (Line 172-) |
| 12. Page 4 line 105: This is more a description of the 'interventions' than 'procedures' | We renamed the Procedures subsection to “Interventions.” |
| 13. Page 4 line 123: Do you mean based on an earlier study?..a study cannot conduct formative research | We conducted formative research before this trial as part of the Ghana EMBRACE Implementation Research Project. We modified the related description to clarify that point, as follows:  “Based on the formative research of the Ghana EMBRACE Implementation Research Project [11,12], B-1 and B-2 were designed to address the relatively low coverage of PNC within two days after delivery.”  (Line 212-) |
| 14. If data from Dodowa was excluded, how was the sample size of 3000 achieved? | We intended to achieve a sample size of 30,000 for mortality assessment by pooling Health and Demographic Surveillance Sites data before, during, and after the trial, even after excluding Dodowa. We modified the limitations provided at the end of the Discussion section to clarify this, as follows:  “Third, the sample used to assess mortality reduction might not have been sufficient to provide accurate numbers, particularly for maternal mortality, since one of the study sites (Dodowa) was excluded from the mortality assessment. Thus, since the number of maternal mortality cases was small, the hypothesis testing was sensitive to small changes in the number of mortality cases.”  (Line 495-) |
| 15. Was this trial registered? | We registered this trial in ISRCTN. We clarified it in the abstract, in addition to the main text.  This cluster randomized controlled trial (ISRCTN: 90618993) was conducted at three Health and Demographic Surveillance Systems Sites in Ghana. (Line 6-)  The study protocol was registered in the International Standard Randomized Controlled Trial Number Registry (90618993).  (Line 300-) |
| 16. Typically, the rule of funder etc comes at the end of the paper not in the methods. PLOS medicine instructions regrading this, comes under the financial disclosure statement which is additional information requested at submission. | We removed the roles of funder statement from the main text. |
| 17. To what extent was the significant increase in coverage of PNC attributed to longer facility stay vs CHW home visits? This has important implications for practice. | We added the result of “PNC within 48 hours of delivery by home visits” to Table 2. We mentioned the improvement in home visits in the Discussion section, in relation to the role of community health workers, as follows:  “The coverage of SBA delivery reached approximately 80% during the trial period. Almost of those who had SBA delivery received PNC within 48 hours. Thus, improvements in the place of delivery and longer retention at a health facility were intensified as the main drivers of increasing CoC completion. During the trial period, 6% of woman-child pairs in the intervention arm and 12% in the control arm received the first PNC within 48 hours by home visits, showing an improvement from being almost zero before the trial period.”  (Line 408-) |
| 18. Page 10 line 315, please rephrase the last sentence to improve clarity. | We modified and added to the content in the last sentence to reflect the two reviewers’ comments, as follows:  “In Ghana, only 21% of deliveries took place at health facilities with comprehensive emergency obstetric care in 2010 [52]. The scope of this intervention did not include improvements in emergency obstetric care or the quality of MNCH services during ANC, delivery, and PNC. Thus, enhancing access to MNCH services should be made in line with an improvement in emergency obstetric care and the quality of MNCH services.”  (Line 466-) |
