# Supplementary material for: Evaluation of a package of continuum of care interventions for improved maternal, newborn, and child health outcomes and service coverage in Ghana: A cluster-randomized trial
Source: PLoS Med. 2021 Jun 25;18(6):e1003663. doi: 10.1371/journal.pmed.1003663 (PMC8232410; doi:10.1371/journal.pmed.1003663)
Supplement: Attachment — Submitted filename: Response_to_reviewer3_R3.docx [file pmed.1003663.s006.docx]

Thank you very much for taking the time to review our manuscript and allow us to make revisions. A point-by-point response to your comments is listed below.

Requests from the editors:

| 1. Please adapt your data statement so that obtaining data via the study principal investigator is not mentioned - this is not consistent with PLOS' data policy: | Thank you very much for instruction. This study used two types of data: 1) face-to-face interview survey data collected by the study team and 2) pregnancy and birth outcome registration data routinely collected by Health Research Centres that managed the Health and Demographic Surveillance Sites. For 1), we will upload the anonymized survey data at <https://fairsharing.org/> upon acceptance of the manuscript. For 2), since it is a third-party data collected by Health Research Centres, we do not have the legal rights to share them openly.  We modified the data access statement as follows:  “The demographic data from the Health and Demographic Surveillance Systems sites are under closed access, but they can be requested following their regulations. The data may be accessible with the permission from the Directors of Navrongo Health Research Centre (P.O. Box 114, Navrongo, Upper East Region, Ghana; director@navrongo-hrc.org and administrator@navrongo-hrc.org) and Kintampo Health Research Centre (P.O. Box 200, Kintampo-North Municipality, Brong Ahafo Region, Ghana; enquiries@kintampo-hrc.org). Moreover, face-to-face interview data can be accessible at <https://fairsharing.org>.” (Lines 540-) |
| --- | --- |

Points from our academic editor:

| 1. According to the trial registration, the authors collected information on intervention coverage, fidelity, cost and sustainability of the implementation. I suspect that the group plans to publish a separate process evaluation, but I believe a minimum in the present paper would be to include information on intervention coverage of the target population, which could be done with a few additional sentences in the results and discussion sections. | We added the following descriptions on the intervention coverage in the Results and Discussion sections.  In the Results section:  “From these health facilities, 83.8% of women in the follow-up survey received the package of CoC interventions.” (Lines 329-)  In the Discussion section:  “In the intervention arm, 83.8% of women and children received the package of CoC interventions.” (Lines 404-) |
| --- | --- |
| 2. The authors do not adhere to WHO's definition of perinatal mortality. They use livebirths as the denominator, whereas WHO (and most publications) use total births (still and live). Please correct the denominator used for perinatal mortality throughout the paper. | We modified the perinatal mortality and stillbirth reporting in Table 3, Fig 2, and the main text by clarifying that the number of live and stillbirth cases is the denominator.  “Perinatal mortality was 130 cases among 6,036 live and stillbirths in the intervention arm and 104 among 4,196 in the control arm during the trial period (p=0.303; In the DiD analysis, perinatal mortality rate decreased by 19% or equivalent to 5 case per 1,000 births) and 71 among 3,399 in the intervention arm and 64 among 2,868 in the control arm after the trial period (p=0.745; In the DiD analysis, perinatal mortality rate decreased by 13% or equivalent to 4 cases per 1,000 births).” (Lines 345-) |

Reviewer #1

| 1. I thank the authors for their consideration of my original points. Their responses and changes to the manuscript are largely acceptable, with one or two minor exceptions. | Thank you very much for your valuable advice in the previous and current revisions. |
| --- | --- |
| 2. In relation to subgroup analyses, the authors have not carried out interaction tests to determine whether the intervention effects are different between subgroups of the population. The analyses are simply stratified, and it is not possible to tell whether the apparent differences are within what might be expected by chance variation. | We modified S1 Table and the Results section to clarify that the analyses in S1 Table presented the estimated coefficients of interaction effects. In the analysis, woman-child pairs were divided into groups that represented the combinations of an area-specific characteristic and arm. Then this group variable was interacted with “the follow-up period” variable to create an interaction term (that is, we did not conduct triple difference-in-differences analysis). In S1 Table, AORs, CIs, and p-interactions are presented for the interaction terms only.  We modified the descriptions of the interaction analyses as below:  “S1 Table presented differences in CoC completion by the characteristics of the nearest health facilities and sub-districts as the results of interaction term analyses. First, woman-child pairs in the control arm were categorized into two groups by living closely to an intervention sub-district or not. Among those living in a control sub-district, the level of improvement in CoC completion was not significantly different from those living in an intervention sub-district if they lived close to an intervention sub-district (AOR for DiD=0.80; 95% CI: 0.43 to 1.48; p=0.479). If woman-child pairs lived in a control sub-district and far from an intervention sub-district, the level of improvement in CoC completion was significantly different from those living in an intervention sub-district (AOR for DiD=0.45; 95% CI: 0.26 to 0.78; p=0.004). Second, woman-child pairs in each arm were categorized into four groups by the types of the nearest health facility. In the intervention arm, woman-child pairs had a higher level of improvement in CoC completion if their nearest facility was CHPS (AOR for DiD=2.12, 95% CI: 1.01 to 4.42; p=0.004) compared to those nearest facility was a public hospital. Third, woman-child pairs in each arm were categorized into three groups by the existence of higher-tier health facilities in a sub-district of living. Compared to those living in a control sub-district with both public hospitals and health centers, woman-child pairs had a higher level of improvement in CoC completion if they lived in an intervention sub-district without public hospitals or health centers (AOR for DiD=5.47, 95% CI: 1.87 to 16.0; p=0.002) and with both public hospitals and health centers (AOR for DiD=11.0, 95% CI: 3.08 to 39.3; p<0.001). Fourth, woman-child pairs in each arm were categorized into two groups by the density of health facilities in their sub-district of living. Compared to those living in a control sub-district that had a lower density of health facility, woman-child pairs had a higher level of improvement in CoC completion if they lived in an intervention sub-district with a higher density of health facilities (AOR for DiD=3.40, 95% CI: 1.75 to 6.50; p<0.001). Fifth, woman-child pairs in each arm were categorized into two groups by the dispatch of midwives in their sub-district of living. Compared to those living in a control sub-district that did not have a midwife, woman-child pairs had a higher level of improvement in CoC completion if they lived in an intervention sub-district that had at least one facility with a midwife (AOR for DiD=1.98, 95% CI: 1.23 to 3.21; p=0.005).” (Line 376-) |
| 3. When talking about mortality outcome, it is not clear what is mean by a X point difference (e.g. line 342). | We clarified that the absolute measurement refers to reductions in mortality ratio or rate; for example, the number of maternal mortality cases reduced per 100,000 live births.  For example, we modified the explanations in the Results section as follows: “In the DiD analysis, maternal mortality ratio increased by 60% or equivalent to 64 cases per 100,000 live births” (Lines 341-) |

Reviewer #3

| 1. Thanks for a much improved manuscript. Just a few mostly grammatical issues. Page 20 line 409, sentence starting with 'Almost' is unclear. Do you mean 'Almost all of those'? | Thank you very much for your appreciation on the revised manuscript. We revised the sentence according to your advice as follows:  “The majority of those who had SBA delivery received PNC within 48 hours. During the trial period, 75% of woman-child pairs in the intervention arm and 68% in the control arm received the first PNC within 48 hours in a health facility.” (Lines 422-) |
| --- | --- |
| 2. Line 411: Do you mean 'identified' rather than 'intensified'? | We modified the sentence as follows:  “Thus, improvements in the place of delivery and longer retention at a health facility were the main drivers of increasing CoC completion.” (Lines 425-) |
| 3. Line 412_ Does this mean that the effect was more in the control arm compared to the intervention arm? Could this be due to the level of contamination in this study? Can we say that for sure? This may need to be clarified in the discussion/conclusion. | In the control arm, infrastructure and human resources in health facilities were largely insufficient to encourage women and children to stay longer at health facilities after delivery. Thus, PNC by home visits would be feasible in control health facilities when health workers there recognized the importance of PNC and CoC. In the intervention arm, the first PNC within 48 hours could be provided at a health facility if women and children were retained there. PNC by home visits would also feasible among small health facilities in the intervention arm without sufficient infrastructure and human resources.  We added the explanation regarding home-based PNC as an alternative to facility-based PNC as follows:  “Home visits are an alternative option of providing PNC, especially when women and children cannot stay longer at a health facility after delivery. In the intervention arm, 14 health facilities among 56 provided B-1 to encourage women and children to stay longer after delivery. The other health facilities did not have sufficient rooms and human resources for B-1. In these health facilities, B-2 is an important option so that women and children could receive PNC at home.” (Lines 426-) |
