# Supplementary material for: Evaluation of a package of continuum of care interventions for improved maternal, newborn, and child health outcomes and service coverage in Ghana: A cluster-randomized trial
Source: PLoS Med. 2021 Jun 25;18(6):e1003663. doi: 10.1371/journal.pmed.1003663 (PMC8232410; doi:10.1371/journal.pmed.1003663)
Supplement: Attachment — Submitted filename: Response to reviewers.docx [file pmed.1003663.s007.docx]

Thank you very much for taking the time to review our manuscript and allow us to submit the revision. A point-by-point response to your comments is listed below.

[Requests from Editors]

1. In your data statement, we suggest adapting the final element to "Moreover, anonymised face-to-face interview data are available at ..." (assuming this is correct). Is there a specific webpage at fairsharing.org that you can quote here?

We stored the face-to-face interview data at the following URL: https://figshare.com/s/26cf77b61d455b4bb7ba.

We followed this advice to add the description in Data Sharing metadata section as follows: “**Moreover, face-to-face interview data can be accessible at https://figshare.com/s/26cf77b61d455b4bb7ba.**”

2. Please adapt the title to "Evaluation of a package of continuum of care ...".

We followed this advice to change the title to “**Evaluation of a package of continuum of care interventions for improved maternal, newborn, and child health outcome and service coverage in Ghana: A cluster-randomized trial.**”

3. At line 3 of your abstract, would "complete" or "comprehensive" be clearer than "completed"?

We followed this advice to modify the sentence as follows: “**In low- and middle-income countries, the continuum of care (CoC) for maternal, newborn, and child health (MNCH) is not always complete.**” (Lines 2-3)

4. Please make that "were lower" at line 29.

We followed this advice to modify the sentence as follows: “**Maternal complications that required hospitalization during pregnancy were lower in the intervention**….” (Lines 28-30)

5. At line 35, please adapt the text slightly to: "As this study was conducted in a real-world setting, possible limitations included differences ...".

We followed this advice to modify the sentence as follows: “**As this study was conducted in a real-world setting, possible limitations included differences in the type and scale of health facilities and the size of sub-districts**…..” (Lines 35-37)

6. At line 43, please make that "evidence of ...".

We followed this advice to modify the sentence as follows: “**It did not find evidence of reduced perinatal and neonatal mortality.**” (Lines 42-43)

7. At line 52, please make that "To our knowledge, no previous study ...".

We followed this advice to modify the sentence as follows: “**To our knowledge, no previous study has evaluated the effectiveness of interventions on CoC completion (or receiving all the key services along the CoC) under real-world settings.**” (Lines 52-54)

8. Please use the active voice in 1-2 points in your Author Summary. We suggest "We conducted a cluster-randomized controlled trial ..." at line 56.

We followed this advice to modify the sentences in “What Did the Researchers Do and Find?” section by using the active voice consistently as follows: (Lines 56-66))

- **We conducted a cluster-randomized controlled trial to evaluate the effectiveness of an integrated package of CoC interventions in a variety of healthcare settings and health facilities at three Health and Demographic Surveillance Systems sites in Ghana.**
- **An integrated package of interventions that aimed at enhancing CoC in MNCH increased CoC completion and decreased maternal complications requiring hospitalization during pregnancy. It also reduced maternal mortality after the trial period, although evidence of decreasing perinatal and neonatal mortality was not found.**
- **We found strong spillover of intervention effectiveness among mothers and children living in sub-districts in the control arm.**

9. At line 294, please make that "Ethical considerations".

We followed this advice to modify the subsection heading as follows: “**Ethical considerations**”

10. Please use the word "thus" just once in the paragraph beginning at line 524.

We followed this advice to modify the first sentence of the paragraph by removing the word “thus,”: “**This study indicates that the level of CoC completion among women and children can be improved in a real-world setting;** …” (Lines 524-525)

11. Please remove the information on funding, competing interests and data sharing from the end of the main text. In the event of publication this will appear in the article metadata, via entries in the submission form.

We followed this advice to remove the subsections for “Declaration of Interests,” “Data sharing,” and “Funding” from the main text.

12. Please use the abbreviation "PLoS ONE" in the reference list.

We followed this advice to modify the reference numbers 5, 6, 11, 13, 20, 38, and 50 by changing PloS One to PloS ONE.

13. Please avoid "ICC=0.000" (in table 5 and any other examples).

We followed this advice to modify the presentation of ICC for “Maternal complications within 6 weeks of delivery which required hospitalization” as “**<0.001**”
