# Supplementary material for: Evaluation of a package of continuum of care interventions for improved maternal, newborn, and child health outcomes and service coverage in Ghana: A cluster-randomized trial
Source: PLoS Med. 2021 Jun 25;18(6):e1003663. doi: 10.1371/journal.pmed.1003663 (PMC8232410; doi:10.1371/journal.pmed.1003663)
Supplement: Peer review history

# Editor report: Decision Letter 0

**DOI:** 10.1371/journal.pmed.1003663.r001

**Authors:** Richard Turner

27 Aug 2020

Dear Dr Jimba, 

Thank you for submitting your manuscript entitled "The effectiveness of a package of continuum of care interventions for improved maternal, newborn, and child health outcome and service coverage in Ghana: A cluster-randomized trial" for consideration by PLOS Medicine.

Your manuscript has now been evaluated by the PLOS Medicine editorial staff and I am writing to let you know that we would like to send your submission out for external assessment.

However, before we can send your manuscript to reviewers, we need you to complete your submission by providing the metadata that is required for full assessment. To this end, please login to Editorial Manager where you will find the paper in the 'Submissions Needing Revisions' folder on your homepage. Please click 'Revise Submission' from the Action Links and complete all additional questions in the submission questionnaire.

Please re-submit your manuscript within two working days, i.e. by .

Login to Editorial Manager here: https://www.editorialmanager.com/pmedicine

Once your full submission is complete, your paper will undergo a series of checks in preparation for peer review. Once your manuscript has passed all checks it will be sent out for review. 

Feel free to email us at plosmedicine@plos.org if you have any queries relating to your submission.

Kind regards,

Richard Turner, PhD

Senior editor, PLOS medicine

rturner@plos.org

# Peer review: Decision Letter 1

**DOI:** 10.1371/journal.pmed.1003663.r002

**Authors:** Richard Turner

6 Oct 2020

Dear Dr. Jimba,

Thank you very much for submitting your manuscript "The effectiveness of a package of continuum of care interventions for improved maternal, newborn, and child health outcome and service coverage in Ghana: A cluster-randomized trial" (PMEDICINE-D-20-04155R1) for consideration at PLOS Medicine. 

Your paper was discussed among the editors and sent to independent reviewers, including a statistical reviewer. The reviews are appended at the bottom of this email and any accompanying reviewer attachments can be seen via the link below:

[LINK]

In light of these reviews, we will not be able to accept the manuscript for publication in the journal in its current form, but we would like to invite you to submit a revised version that addresses the reviewers' and editors' comments fully. You will appreciate that we cannot make a decision about publication until we have seen the revised manuscript and your response, and we expect to seek re-review by one or more of the reviewers. 

In revising the manuscript for further consideration, your revisions should address the specific points made by each reviewer and the editors. Please also check the guidelines for revised papers at http://journals.plos.org/plosmedicine/s/revising-your-manuscript for any that apply to your paper. In your rebuttal letter you should indicate your response to the reviewers' and editors' comments, the changes you have made in the manuscript, and include either an excerpt of the revised text or the location (eg: page and line number) where each change can be found. Please submit a clean version of the paper as the main article file; a version with changes marked should be uploaded as a marked up manuscript.

In addition, we request that you upload any figures associated with your paper as individual TIF or EPS files with 300dpi resolution at resubmission; please read our figure guidelines for more information on our requirements: http://journals.plos.org/plosmedicine/s/figures. While revising your submission, please upload your figure files to the PACE digital diagnostic tool, https://pacev2.apexcovantage.com/. PACE helps ensure that figures meet PLOS requirements. To use PACE, you must first register as a user. Then, login and navigate to the UPLOAD tab, where you will find detailed instructions on how to use the tool. If you encounter any issues or have any questions when using PACE, please email us at PLOSMedicine@plos.org.

We hope to receive your revised manuscript by Oct 26 2020 11:59PM. Please email us (plosmedicine@plos.org) if you have any questions or concerns.

***Please note while forming your response, if your article is accepted, you may have the opportunity to make the peer review history publicly available. The record will include editor decision letters (with reviews) and your responses to reviewer comments. If eligible, we will contact you to opt in or out.***

We ask every co-author listed on the manuscript to fill in a contributing author statement, making sure to declare all competing interests. If any of the co-authors have not filled in the statement, we will remind them to do so when the paper is revised. If all statements are not completed in a timely fashion this could hold up the re-review process. If new competing interests are declared later in the revision process, this may also hold up the submission. Should there be a problem getting one of your co-authors to fill in a statement we will be in contact. YOU MUST NOT ADD OR REMOVE AUTHORS UNLESS YOU HAVE ALERTED THE EDITOR HANDLING THE MANUSCRIPT TO THE CHANGE AND THEY SPECIFICALLY HAVE AGREED TO IT. You can see our competing interests policy here: http://journals.plos.org/plosmedicine/s/competing-interests.

Please use the following link to submit the revised manuscript: 

https://www.editorialmanager.com/pmedicine/

Your article can be found in the "Submissions Needing Revision" folder. 

To enhance the reproducibility of your results, we recommend that you deposit your laboratory protocols in protocols.io, where a protocol can be assigned its own identifier (DOI) such that it can be cited independently in the future. For instructions see http://journals.plos.org/plosmedicine/s/submission-guidelines#loc-methods.

Please ensure that the paper adheres to the PLOS Data Availability Policy (see http://journals.plos.org/plosmedicine/s/data-availability), which requires that all data underlying the study's findings be provided in a repository or as Supporting Information. For data residing with a third party, authors are required to provide instructions with contact information for obtaining the data. PLOS journals do not allow statements supported by "data not shown" or "unpublished results." For such statements, authors must provide supporting data or cite public sources that include it. 

Please let me know if you have any questions. Otherwise, we look forward to receiving your revised manuscript in due course. 

Sincerely,

Richard Turner, PhD

Senior Editor, PLOS Medicine

rturner@plos.org

-----------------------------------------------------------

Requests from the editors:

To your data statement, for readers interested in inquiring about access to study data please add contact details for a non-author contact.

In the abstract and throughout the paper, please add p values alongside 95% CI, where available. 

Please add summary details to your abstract of the number of sites, women and women-child pairs studied, and if possible aggregate demographic details for the participants. 

Where you state the study dates in the abstract, we suggest adding additional details of the before and subsequent survey periods, along with the inclusion criteria.

Please combine the "methods" and "findings" subsections of your abstract. The final sentence of the new combined subsection should quote 2-3 of the study's main limitations. 

Please state the study's primary and other outcomes early in the "methods and findings" subsection of your abstract.

Please begin the "Conclusions" subsection of your abstract with "In this study, we found that ..." or similar. 

After the abstract, please add a new and accessible "author summary" section in non-identical prose. You may find it helpful to consult one or two recent research papers published in PLOS Medicine to get a sense of the preferred style. 

Please remove the "Role of the funding source" section from the end of the methods section. In the event of publication, information on funding will appear in the article metadata, via entries in the submission form. Similarly, please remove information on competing interests from the end of the main text.

Throughout the text, please ensure that reference call-outs fall consistently before punctuation, and remove spaces from within the square brackets (e.g., "... (LMICs) [1,2].").

Thank you for including a completed CONSORT checklist. Please adapt the checklist so that individual items are referred to by section (e.g., "Methods") and paragraph number rather than by line or page numbers, as the latter generally change in the event of publication. 

Please remove the published study protocol from the supplementary files - a reference will suffice. 

Comments from the reviewers:

*** Reviewer #1: 

Alex McConnachie, Statistical Review

Shibanuma et al present a report on a large cluster randomised trial of a package of health service interventions to improve the continuity of care for pregnant women and their babies in Ghana. This review considers the use of statistics in the paper.

Overall, the study design, statistical analyses, their presentation and interpretation, are good. I do have a number of observations, however. Whist they are many, each of these is relatively minor, and if dealt with, should improve the paper.

According to the protocol paper, the primary outcome is the CoC completion rate, but this paper does not specify which outcome is primary. Since this was prespecified, it should really be presented first. The abstract, and the results section, both present mortality first.

Also in the abstract, it is noted that perinatal and neonatal mortality were not significantly reduced in the intervention arm, but AORs and CIs are not provided - should they be?

The fact that sub-districts that contained a district hospital were automatically allocated to the intervention group raised some concerns when I first read it, but after reading the discussion, I can see that this was perhaps a necessary decision. I think the rationale for doing this should be stated in the methods, and not left until the discussion. Also, I am guessing that this relates to 3 intervention and 3 control sub-districts (since they say there was one district hospital per district), but it would help if the authors were absolutely clear on how many sub-districts were affected.

The sample size sections could be better. Firstly, they are in the wrong order, if CoC completion was the primary outcome. Secondly, they do not give the assumed average cluster size, or whether the calculation assumes equal cluster sizes or allows for variable cluster sizes. Thirdly, the protocol paper states that the required sample size for the mortality analysis was 15,000, not 30,000 as stated in this paper.

In the statistical methods, chi-square tests are reportedly used to compare outcomes, but this is not suitable for a cluster randomised trial. Reporting analyses without adjustment for covariates is fine, but any analysis of a cluster randomised trial should account for the clustering. The mixed effects logistic regression analysis is more appropriate. However, the protocol paper states that a GEE method was to be used, so the authors should justify their decision to use an alternative method.

Also reported in the protocol paper is that the analyses will be by intention to treat, and I think that is worth adding to this paper. The protocol paper states that all randomised participants will be included in analyses, regardless of missing data. Whilst in my opinion, this is not a requirement of ITT, it is nevertheless a worthwhile thing to do. However, it should be made clear that this has been done, and what assumptions or specific methods were used to deal with missing outcome data. If, on the other hand, this was not done, then the authors should again justify this deviation from their original plan.

The mortality outcomes were collected in three phases (before, during and after the trial), but no mention is made of the analysis of these outcomes during the trial, other than in the tables; in the main text of the paper, only the post-trial period mortality results are reported. Also, in the footnote to Table 4, it seems as though each post-randomisation time point was analysed separately. Would it be better to analyse both time points within the same regression model, i.e. pooling data from three time points, modelling the time effect as a 3-level categorical variable, and estimating the intervention effects at each time point through the addition of time-by-intervention interactions?

The analyses presented in Supplementary Table 1 seem odd. I do not see the value of comparing those who live in or close to an intervention area, with those living in a control area but not close to an intervention area; it would make more sense to consider the 3-level categorical variable of living in an intervention area vs. living close to an intervention area vs. not living close to an intervention area. When looking at the type of the nearest facility, all facilities in control areas are treated the same. The pattern of differences between types of facility seen in intervention areas may be seen in control areas as well, but it is not possible to tell from the analysis that is presented. The third analysis breaks down the intervention sub-districts according to the type of interventions available; it is not possible to split control group sub-districts in the same way, so this analysis makes more sense.

The remainder of Supplementary Table 1 is different, in that it is looking at intervention effects within subgroups of sub-districts. However, these analyses would be improved if they used interaction terms to test for differences in intervention effects between subgroups. It is not sufficient to estimate intervention effects within subgroups and draw conclusions form which are statistically significant. Statistical evidence of different intervention effects between subgroups is required to draw any conclusions.

In Figure 2, for the mortality outcomes, does it make sense to state the number of women or mother-child pairs who were "surveyed"? No one was surveyed, if the data were taken from administrative records. Also, if these data were only available in two of the three HDSS sites involved in the study, is it fair to say that 32 sub-districts were included in these analyses?

The footnote of Table 2 states that chi-square tests were used to compare means between arms. This is not correct - a chi-square test compares proportions or percentages between groups, though as stated above, simple chi-square tests are not appropriate for a cluster randomised trial.

The footnote to Table 4 reports that the models were adjusted for several covariates. It is slightly surprising that so much information is available from administrative records, and that it is complete. I would assume a fairly high level of missingness in administrative data - if that is the case, how much missing data was there, and how was it dealt with?

In the CONSORT checklist, I note that the ICCs for the data presented in the paper are not provided. Also, the checklist suggests reporting both relative and absolute effect sizes; was this done? As far as I can tell, the results are exclusively reported as relative effects.

*** Reviewer #2: 

Thank you for this manuscript, which describes an important study to look at the effectiveness of intervention to support completion of the continuum of care for mothers and children in a selection of health facilities in Ghana. 

As a reviewer, I am not qualified to scrutinise the statistics and methodological approaches applied and would ask that an appropriate reviewer with strong epi or med-stats background offers their comment on this article. 

Some minor changes to English language would improve readability. Some specific areas of note as follows:

Background section:

p1 line 24: suggest rephrase - sentence doesn't read very easily

p1 link 26-28: suggest use the word "however" instead of while

p2 line 45-48 : Sentence is too long - suggest to break up.

Methods: 

p4 first few paragraphs on district hospitals - the description of this is a bit convoluted and does bring into question the randomisation process overall. I think this is then well-described in the discussion, but still a bit confusing here.

Results section:

The first paragraph of the results is difficult to read and unclear.

p7 line 221-225

Some confusing expressions here - I think less in the formal marriage refers to someone who is either unmarried or single - correct?

In the health insurance - more likely to have health insurance?

In the discussion would be helpful to have some discussion about how this project links to other efforts to scale up emergency obstetric care and postnatal care of newborns. 

*** Reviewer #3: 

Thanks for submitting a very important trial of a bundle of interventions to reduce maternal and perinatal morbidity and mortality. I have a few comments that may be useful in improving the manuscript.

Abstract

Control group is not defined

Conclusions could be boldly linked to results

Introduction

Page 1: Line 15: Consider 'give birth' rather than 'deliver newborns'

How was the pre-pregnancy component of COC measured in your study?

Page 1: Line 24-25: This clain is not accurate. See systematic review published in 2016:

Kikuchi K, Okawa S, Zamawe COF, Shibanuma A, Nanishi K, Iwamoto A, et al. (2016) Effectiveness of Continuum of Care—Linking Pre-Pregnancy Care and Pregnancy Care to Improve Neonatal and Perinatal Mortality: A Systematic Review and Meta-Analysis. PLoS ONE 11(10): e0164965. https://doi.org/10.1371/journal.pone.0164965

Page 1 line 29: Global MMR target is 70/100, 000 but no country should be more than 140/100, 000., so this statement as it ideas not give a true picture of how much behind Ghana is. Either compare with the country level target of 140, 000/100, 000 or compare the Ghanaian figure with Africa regional average.

Page 1, line 31: Coverage of PNC is unclear, is this 2 visits, 1 visit? If the coverage was based on a visit within 24 hours, say so.

Page 2 line 35, please rephrase to improve clarity. Line 36 these coverage refers to one visit within 24 hours? Is this feasible? Do Ghana CMWs visit homes within 24hours of birth? Please rephrase line 35-38, so that your message is factually correct.

Page 2 line 41 to 49 should be in the methods, not introduction. The interventions should be better described in the methods.

The CoC card is essentially a one page record of CoC intervention received including health education? If this is correct, please rephrase to accurately describe what the CoC card is and what it was used for.

Page 3, line 68-74 typically this comes last in the methods, just before the results section.

Page 4: The 32 sub-districts were in how many districts? The randomisation process, intervention and control clusters is very unclear. It will be difficult to replicate this study with the information provided.

Page 4 line 105: This is more a description of the 'interventions' than 'procedures'

Page 4 line 123: Do you mean based on an earlier study?..a study cannot conduct formative research

If data from Dodowa was excluded, how was the sample size of 3000 achieved? 

Was this trial registered?

Typically, the rule of funder etc comes at the end of the paper not in the methods. PLOS medicine instructions regrading this, comes under the financial disclosure statement which is additional information requested at submission.

To what extent was the significant increase in coverage of PNC attributed to longer facility stay vs CHW home visits? This has important implications for practice.

Page 10 line 315, please rephrase the last sentence to improve clarity.

***

Any attachments provided with reviews can be seen via the following link:

[LINK]

# Author comment: Author response to Decision Letter 1

**DOI:** 10.1371/journal.pmed.1003663.r003

5 Nov 2020

Attachment Submitted filename: Response_to_reviewer_v06.docx Click here for additional data file.

# Peer review: Decision Letter 2

**DOI:** 10.1371/journal.pmed.1003663.r004

**Authors:** Emma Veitch

11 Dec 2020

Dear Dr. Jimba,

Thank you very much for submitting your revised paper "The effectiveness of a package of continuum of care interventions for improved maternal, newborn, and child health outcome and service coverage in Ghana: A cluster-randomized trial" (PMEDICINE-D-20-04155R2) for consideration at PLOS Medicine. 

The revisions were seen again by two of the previous reviewers and by the academic editor, as well as being discussed amongst the in-house editors. The reviews, along with comments from the academic editor and inhouse editors, are enclosed below and I hope you find their comments constructive. 

Given the review comments we don't feel we can offer publication at this point but would like to invite you to revise further and we should be able to make a decision on the next version; it's possible that the authors' revision will need to be re-reviewed externally. 

In revising the manuscript for further consideration, your revisions should address the specific points made by each reviewer and the editors. Please also check the guidelines for revised papers at http://journals.plos.org/plosmedicine/s/revising-your-manuscript for any that apply to your paper. In your rebuttal letter you should indicate your response to the reviewers' and editors' comments, the changes you have made in the manuscript, and include either an excerpt of the revised text or the location (eg: page and line number) where each change can be found. Please submit a clean version of the paper as the main article file; a version with changes marked should be uploaded as a marked up manuscript.

In addition, we request that you upload any figures associated with your paper as individual TIF or EPS files with 300dpi resolution at resubmission; please read our figure guidelines for more information on our requirements: http://journals.plos.org/plosmedicine/s/figures. While revising your submission, please upload your figure files to the PACE digital diagnostic tool, https://pacev2.apexcovantage.com/. PACE helps ensure that figures meet PLOS requirements. To use PACE, you must first register as a user. Then, login and navigate to the UPLOAD tab, where you will find detailed instructions on how to use the tool. If you encounter any issues or have any questions when using PACE, please email us at PLOSMedicine@plos.org.

We expect to receive your revised manuscript by Jan 01 2021 11:59PM. Please email us (plosmedicine@plos.org) if you have any questions or concerns.

***Please note while forming your response, if your article is accepted, you may have the opportunity to make the peer review history publicly available. The record will include editor decision letters (with reviews) and your responses to reviewer comments. If eligible, we will contact you to opt in or out.***

We ask every co-author listed on the manuscript to fill in a contributing author statement, making sure to declare all competing interests. If any of the co-authors have not filled in the statement, we will remind them to do so when the paper is revised. If all statements are not completed in a timely fashion this could hold up the re-review process. If new competing interests are declared later in the revision process, this may also hold up the submission. Should there be a problem getting one of your co-authors to fill in a statement we will be in contact. YOU MUST NOT ADD OR REMOVE AUTHORS UNLESS YOU HAVE ALERTED THE EDITOR HANDLING THE MANUSCRIPT TO THE CHANGE AND THEY SPECIFICALLY HAVE AGREED TO IT. You can see our competing interests policy here: http://journals.plos.org/plosmedicine/s/competing-interests.

Please use the following link to submit the revised manuscript: 

https://www.editorialmanager.com/pmedicine/

Your article can be found in the "Submissions Needing Revision" folder. 

To enhance the reproducibility of your results, we recommend that you deposit your laboratory protocols in protocols.io, where a protocol can be assigned its own identifier (DOI) such that it can be cited independently in the future. For instructions see http://journals.plos.org/plosmedicine/s/submission-guidelines#loc-methods.

Please ensure that the paper adheres to the PLOS Data Availability Policy (see http://journals.plos.org/plosmedicine/s/data-availability), which requires that all data underlying the study's findings be provided in a repository or as Supporting Information. For data residing with a third party, authors are required to provide instructions with contact information for obtaining the data. PLOS journals do not allow statements supported by "data not shown" or "unpublished results." For such statements, authors must provide supporting data or cite public sources that include it. 

We look forward to receiving your revised manuscript. 

Sincerely,

Emma Veitch, PhD

PLOS Medicine

On behalf of Richard Turner, PhD, Senior Editor, 

PLOS Medicine

plosmedicine.org

-----------------------------------------------------------

Requests from the editors:

*Please adapt your data statement so that obtaining data via the study principal investigator is not mentioned - this is not consistent with PLOS' data policy: https://journals.plos.org/plosmedicine/s/data-availability.

Points from our academic editor:

According to the trial registration, the authors collected information on intervention coverage, fidelity, cost and sustainability of the implementation. I suspect that the group plans to publish a separate process evaluation, but I believe a minimum in the present paper would be to include information on intervention coverage of the target population, which could be done with a few additional sentences in the results and discussion sections.

The authors do not adhere to WHO's definition of perinatal mortality. They use livebirths as the denominator, whereas WHO (and most publications) use total births (still and live). Please correct the denominator used for perinatal mortality throughout the paper.

-----------------------------------------------------------

Comments from the reviewers:

Reviewer #1: 

Alex McConnachie, Statistical Review

I thank the authors for their consideration of my original points. Their responses and changes to the manuscript are largely acceptable, with one or two minor exceptions.

In relation to subgroup analyses, the authors have not carried out interaction tests to determine whether the intervention effects are different between subgroups of the population. The analyses are simply stratified, and it is not possible to tell whether the apparent differences are within what might be expected by chance variation.

When talking about mortality outcome, it is not clear what is mean by a X point difference (e.g. line 342).

-----------------------------------------------------------

Reviewer #3: 

Thanks for a much improved manuscript.

Just a few mostly grammatical issues

Page 20 line 409, sentence starting with 'Almost' is unclear. Do you mean 'Almost all of those'?

Line 411: Do you mean 'identified' rather than 'intensified'?

Line 412_ Does this mean that the effect was more in the control arm compared to the intervention arm? Could this be due to the level of contamination in this study? Can we say that for sure? This may need to be clarified in the discussion/conclusion.

all the best

-----------------------------------------------------------

Any attachments provided with reviews can be seen via the following link:

[LINK]

# Author comment: Author response to Decision Letter 2

**DOI:** 10.1371/journal.pmed.1003663.r005

1 Jan 2021

Attachment Submitted filename: Response_to_reviewer3_R3.docx Click here for additional data file.

# Editor report: Decision Letter 3

**DOI:** 10.1371/journal.pmed.1003663.r006

**Authors:** Richard Turner

13 May 2021

Dear Dr. Jimba,

Thank you very much for re-submitting your manuscript "The effectiveness of a package of continuum of care interventions for improved maternal, newborn, and child health outcome and service coverage in Ghana: A cluster-randomized trial" (PMEDICINE-D-20-04155R3) for consideration at PLOS Medicine. We do apologize for the delay in sending you a response. 

I have discussed the paper with editorial colleagues and our academic editor and I am pleased to tell you that, provided the remaining editorial and production issues are dealt with, we expect to be able to accept the paper for publication in the journal.

The remaining issues that need to be addressed are listed at the end of this email. Any accompanying reviewer attachments can be seen via the link below. Please take these into account before resubmitting your manuscript:

[LINK]

***Please note while forming your response, if your article is accepted, you may have the opportunity to make the peer review history publicly available. The record will include editor decision letters (with reviews) and your responses to reviewer comments. If eligible, we will contact you to opt in or out.***

In revising the manuscript for further consideration here, please ensure you address the specific points made by each reviewer and the editors. In your rebuttal letter you should indicate your response to the reviewers' and editors' comments and the changes you have made in the manuscript. Please submit a clean version of the paper as the main article file. A version with changes marked must also be uploaded as a marked up manuscript file.

Please also check the guidelines for revised papers at http://journals.plos.org/plosmedicine/s/revising-your-manuscript for any that apply to your paper. If you haven't already, we ask that you provide a short, non-technical Author Summary of your research to make findings accessible to a wide audience that includes both scientists and non-scientists. The Author Summary should immediately follow the Abstract in your revised manuscript. This text is subject to editorial change and should be distinct from the scientific abstract.

We hope to receive your revised manuscript within 1 week. Please email us (plosmedicine@plos.org) if you have any questions or concerns.

We ask every co-author listed on the manuscript to fill in a contributing author statement. If any of the co-authors have not filled in the statement, we will remind them to do so when the paper is revised. If all statements are not completed in a timely fashion this could hold up the re-review process. Should there be a problem getting one of your co-authors to fill in a statement we will be in contact. YOU MUST NOT ADD OR REMOVE AUTHORS UNLESS YOU HAVE ALERTED THE EDITOR HANDLING THE MANUSCRIPT TO THE CHANGE AND THEY SPECIFICALLY HAVE AGREED TO IT.

Please ensure that the paper adheres to the PLOS Data Availability Policy (see http://journals.plos.org/plosmedicine/s/data-availability), which requires that all data underlying the study's findings be provided in a repository or as Supporting Information. For data residing with a third party, authors are required to provide instructions with contact information for obtaining the data. PLOS journals do not allow statements supported by "data not shown" or "unpublished results." For such statements, authors must provide supporting data or cite public sources that include it.

To enhance the reproducibility of your results, we recommend that you deposit your laboratory protocols in protocols.io, where a protocol can be assigned its own identifier (DOI) such that it can be cited independently in the future. Additionally, PLOS ONE offers an option to publish peer-reviewed clinical study protocols. Read more information on sharing protocols at https://plos.org/protocols?utm_medium=editorial-email&utm_source=authorletters&utm_campaign=protocols

Please review your reference list to ensure that it is complete and correct. If you have cited papers that have been retracted, please include the rationale for doing so in the manuscript text, or remove these references and replace them with relevant current references. Any changes to the reference list should be mentioned in the rebuttal letter that accompanies your revised manuscript.

Please note, when your manuscript is accepted, an uncorrected proof of your manuscript will be published online ahead of the final version, unless you've already opted out via the online submission form. If, for any reason, you do not want an earlier version of your manuscript published online or are unsure if you have already indicated as such, please let the journal staff know immediately at plosmedicine@plos.org.

Please let me know if you have any questions, and we look forward to receiving the revised manuscript.   

Sincerely,

Richard Turner PhD

Senior Editor, PLOS Medicine

rturner@plos.org

------------------------------------------------------------

Requests from Editors:

In your data statement, we suggest adapting the final element to "Moreover, anonymised face-to-face interview data are available at ..." (assuming this is correct). Is there a specific webpage at fairsharing.org that you can quote here?

Please adapt the title to "Evaluation of a package of continuum of care ...". 

At line 3 of your abstract, would "complete" or "comprehensive" be clearer than "completed"?

Please make that "were lower" at line 29.

At line 35, please adapt the text slightly to: "As this study was conducted in a real-world setting, possible limitations included differences ...".

At line 43, please make that "evidence of ...".

At line 52, please make that "To our knowledge, no previous study ...".

Please use the active voice in 1-2 points in your Author Summary. We suggest "We conducted a cluster-randomized controlled trial ..." at line 56. 

At line 294, please make that "Ethical considerations".

Please use the word "thus" just once in the paragraph beginning at line 524.

Please remove the information on funding, competing interests and data sharing from the end of the main text. In the event of publication this will appear in the article metadata, via entries in the submission form. 

Please use the abbreviation "PLoS ONE" in the reference list.

Please avoid "ICC=0.000" (in table 5 and any other examples). 

***

# Author comment: Author response to Decision Letter 3

**DOI:** 10.1371/journal.pmed.1003663.r007

19 May 2021

Attachment Submitted filename: Response to reviewers.docx Click here for additional data file.

# Editor report: Decision Letter 4

**DOI:** 10.1371/journal.pmed.1003663.r008

**Authors:** Richard Turner

20 May 2021

Dear Dr Jimba, 

On behalf of my colleagues and the Academic Editor, Dr Persson, I am pleased to inform you that we have agreed to publish your manuscript "Evaluation of a package of continuum of care interventions for improved maternal, newborn, and child health outcome and service coverage in Ghana: A cluster-randomized trial" (PMEDICINE-D-20-04155R4) in PLOS Medicine.

Before your manuscript can be formally accepted you will need to complete some formatting changes, which you will receive in a follow up email. Please be aware that it may take several days for you to receive this email; during this time no action is required by you. Once you have received these formatting requests, please note that your manuscript will not be scheduled for publication until you have made the required changes.

Prior to final acceptance, fig 1 (CoC card) will need to be removed, as copyright material cannot be published under the CC-BY licence. 

Please also confirm that the data at figshare (which we were unable to access) is fully de-identified. 

In the meantime, please log into Editorial Manager at http://www.editorialmanager.com/pmedicine/, click the "Update My Information" link at the top of the page, and update your user information to ensure an efficient production process. 

PRESS

We frequently collaborate with press offices. If your institution or institutions have a press office, please notify them about your upcoming paper at this point, to enable them to help maximise its impact. If the press office is planning to promote your findings, we would be grateful if they could coordinate with medicinepress@plos.org. If you have not yet opted out of the early version process, we ask that you notify us immediately of any press plans so that we may do so on your behalf.

We also ask that you take this opportunity to read our Embargo Policy regarding the discussion, promotion and media coverage of work that is yet to be published by PLOS. As your manuscript is not yet published, it is bound by the conditions of our Embargo Policy. Please be aware that this policy is in place both to ensure that any press coverage of your article is fully substantiated and to provide a direct link between such coverage and the published work. For full details of our Embargo Policy, please visit http://www.plos.org/about/media-inquiries/embargo-policy/.

To enhance the reproducibility of your results, we recommend that you deposit your laboratory protocols in protocols.io, where a protocol can be assigned its own identifier (DOI) such that it can be cited independently in the future. Additionally, PLOS ONE offers an option to publish peer-reviewed clinical study protocols. Read more information on sharing protocols at https://plos.org/protocols?utm_medium=editorial-email&utm_source=authorletters&utm_campaign=protocols

Thank you again for submitting to PLOS Medicine. We look forward to publishing your paper. 

Sincerely, 

Richard Turner, PhD 

Senior Editor, PLOS Medicine

rturner@plos.org
